# Spatial Visualization of A-to-I Editing in Cells Using Endonuclease V Immunostaining Assay (EndoVIA)

**DOI:** 10.1021/acscentsci.4c00444

**Published:** 2024-07-08

**Authors:** Alexandria L. Quillin, Benoît Arnould, Steve D. Knutson, Jennifer M. Heemstra

**Affiliations:** † Department of Chemistry, Washington University in St. Louis, St. Louis, Missouri 63130, United States; ‡ Merck Center for Catalysis, Princeton University, Princeton, New Jersey 08544, United States; § Department of Chemistry, Princeton University, Princeton, New Jersey 08544, United States

## Abstract

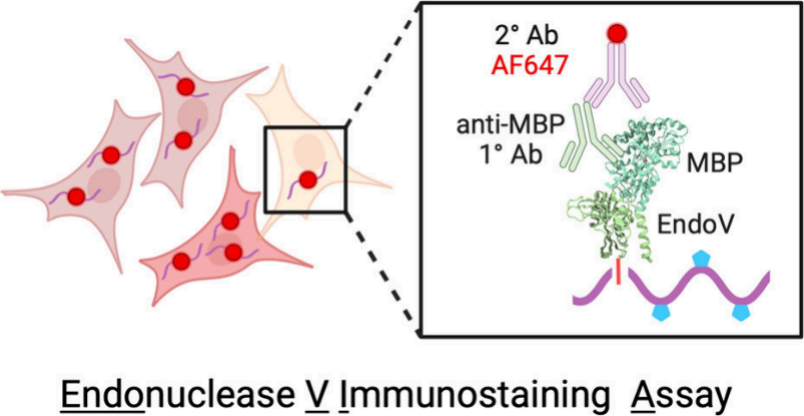

Adenosine-to-inosine (A-to-I) editing is one of the most widespread post-transcriptional RNA modifications and is catalyzed by adenosine deaminases acting on RNA (ADARs). Varying across tissue types, A-to-I editing is essential for numerous biological functions, and dysregulation leads to autoimmune and neurological disorders, as well as cancer. Recent evidence has also revealed a link between RNA localization and A-to-I editing, yet understanding of the mechanisms underlying this relationship and its biological impact remains limited. Current methods rely primarily on *in vitro* characterization of extracted RNA that ultimately erases subcellular localization and cell-to-cell heterogeneity. To address these challenges, we have repurposed endonuclease V (EndoV), a magnesium-dependent ribonuclease that cleaves inosine bases in edited RNA, to selectively bind and detect A-to-I edited RNA in cells. The work herein introduces an endonuclease V immunostaining assay (EndoVIA), a workflow that provides spatial visualization of edited transcripts, enables rapid quantification of overall inosine abundance, and maps the landscape of A-to-I editing within the transcriptome at the nanoscopic level.

## Abstract 2

EndoVIA detects adenosine-to-inosine editing in cells, providing spatial visualization and quantification of overall inosine abundance with nanoscale resolution.

## Introduction

**Figure 1:**
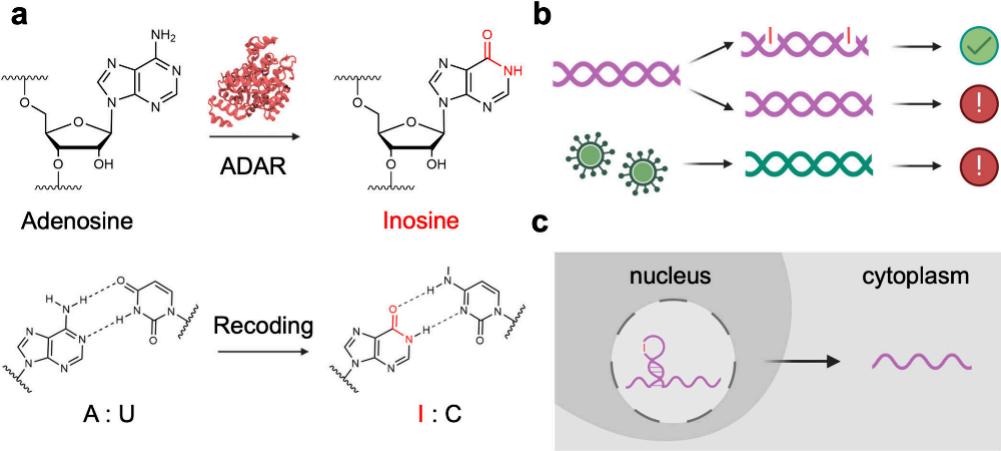
A-to-I editing impacts essential biological functions. (a) ADAR enzymes catalyze adenosine-to-inosine RNA editing, recoding edited sites to be read as guanosine to cellular machinery. (b) Loss of A-to-I editing in endogenous RNAs triggers the innate immune response, synonymous to viral infections. (c) Presence of extensively edited 3′ UTR in mRNA results in nuclear retention to paraspeckles.

Adenosine-to-inosine (A-to-I) editing is one of the most widespread RNA modifications in metazoans and is catalyzed by adenosine deaminases acting on RNA (ADARs).^1^ Among the three ADAR genes encoded in vertebrates (*ADAR1*, *ADAR2*, and *ADAR3*), ADAR1 is ubiquitously expressed and is responsible for the majority of editing in cells.^2,3^ ADAR1 is expressed in a p110 isoform that primarily resides in the nucleus, in addition to an IFN-inducible p150 isoform that localizes to the cytoplasm. Adenosine deamination results in a change of hydrogen bonding such that the resulting inosine base pairs with cytidine, effectively recoding the site to be recognized as guanosine by cellular machinery (Figure 1a).^2^ Therefore, editing that occurs in protein-coding regions of mRNA can lead to multiple protein isoforms and altered function.^2^ While A-to-I editing is most abundant in noncoding and repetitive regions of mRNA and impacts transcript stability and localization,^2,4^ noncoding RNAs such as small-interfering RNAs (siRNAs) and microRNAs (miRNAs) are also edited, affecting global gene expression and cellular functionality.^5,6^ Collectively, A-to-I editing has proven essential for stem cell differentiation, embryogenesis, brain development, and cellular immunity.^2,7,8^

ADAR1 is believed to play a critical role in regulating the activation of innate cellular immunity by targeting double-stranded RNA (dsRNA) made from long, inverted Alu repetitive elements that are located within introns and untranslated regions.^9^ These embedded Alu repeats make up 10% of the human genome and create ∼300 base pair long RNAs when transcribed that look very similar to foreign, viral RNA. ADAR prevents the activation of the cytosolic innate immune system by editing these endogenous dsRNAs and marking them as “self” (Figure 1b). Given the significant role A-to-I editing plays in maintaining cellular function, it is unsurprising that dysregulation has been linked to multiple neurological disorders, autoimmune disorders, and cancers.^6,10,11^ A-to-I editing levels can display hyper- or hypo-editing depending on the specific cancer type.^12,13^ Notably, knockout of ADAR1 has been shown to be lethal in cell and animal models, and ADAR1 inhibition shows significant promise as a therapeutic strategy for treating cancer.^9,14−20^ Moreover, it has been observed that in melanoma an A-to-I editing deficiency directly contributes to the melanoma metastatic phenotype.^21^ Collectively, these findings along with others highlight a clear link between A-to-I editing and the disease state, while speaking to the intricate relationship between editing and cancer that has yet to be fully elucidated. Leveraging this connection toward diagnostics and therapeutics will require greater understanding of the relationship between editing and disease progression, which has yet to be achieved with currently available methods.

The significance of A-to-I editing extends beyond sheer quantity; its influence on RNA localization is equally pivotal. Extensive A-to-I editing of inverted Alu repeats located within the 3′ UTR of mRNAs plays an essential role in nuclear retention,^22−25^ and the presence or absence of an edited 3′ UTR has been demonstrated to drastically alter RNA subcellular localization (Figure 1c). One example of this change in RNA localization was observed by Chen et al. with the human gene *Nicolin 1*. Nicolin 1 is expressed in multiple isoforms, but strikingly the only isoform that remains within the nucleus features an extensively edited, inverted Alu repeat in its 3′ UTR.^24^ Beyond Alu repeats, the 3′ UTRs of RNAs also contain *cis*-regulatory elements, often referred to as “zipcodes”, that influence the localization of mRNA.^26,27^ These sequences serve as encoded cellular addresses that RNA-binding proteins (RBPs) interact with to facilitate trafficking.^28^ Considering that A-to-I editing primarily occurs within these noncoding regions, mRNA localization is subject to change based on the extent of editing. These examples underscore the profound connection between A-to-I editing and RNA localization. However, these findings also represent the extent of our current knowledge of the A-to-I editing landscape due to the lack of tools capable of mapping inosine within cells.

**Figure 2:**
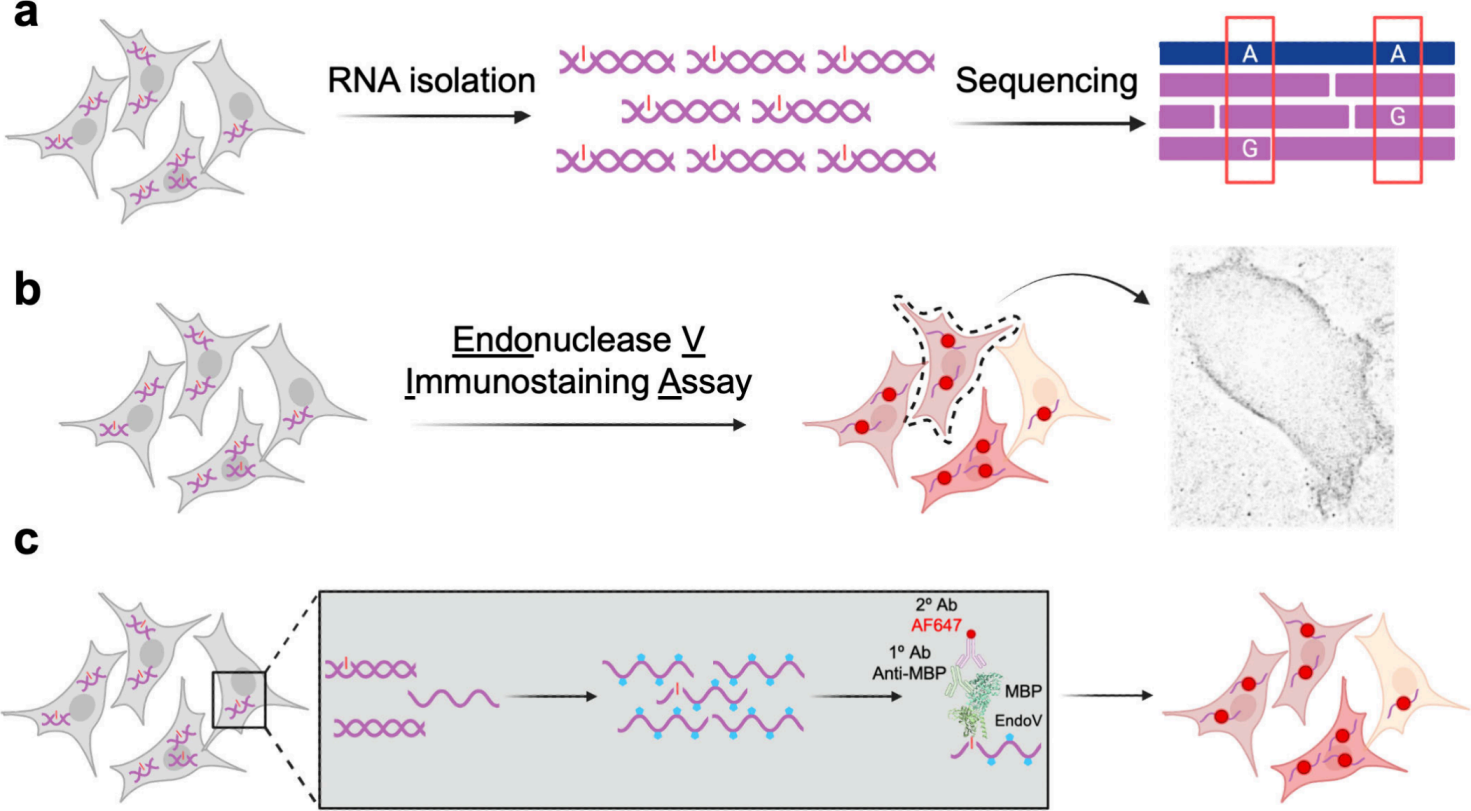
Detection of inosine-containing RNA in cells using EndoVIA. (a) RNA is isolated from cells and then sequenced to identify A–G transitions between RNA sequencing reads (purple) and a reference genome (blue), thus mapping A-to-I edited sites. (b) EndoVIA detects cell-to-cell variation in A-to-I editing and localization of edited transcripts at the nanoscopic level. (c) EndoVIA workflow; cells are fixed, treated with glyoxal (blue pentagons) to denature RNA secondary structure, prepped, and stained using EndoV and a series of antibodies to detect A-to-I edited sites *in situ*.

The most widespread technique used to characterize A-to-I editing is RNA sequencing (RNA-seq), in which RNA is extracted from cells, pooled, and subjected to high-throughput sequencing. Resulting sequences are compared to a reference genome, and edited sites are identified from A–G transitions.^5^ While it remains the gold standard for identifying edited transcripts, unfortunately RNA sequencing along with other common methods requires RNA extraction that ultimately erases subcellular localization (Figure 2a). Extraction also precludes the opportunity to compare cell-to-cell variation *in situ*, which is a hallmark of tumor heterogeneity.

Traditional cellular RNA imaging techniques, such as fluorescence *in situ* hybridization (FISH) and genetically encodable tags that bind fluorescent proteins or dyes, have revolutionized the way RNA is visualized in cells.^29−31^ However, these approaches alone cannot detect editing status and lack the sensitivity required to discern single-nucleotide modifications. As a result, specialized methods have been developed to image the localization of RNA modifications. Some of the first examples are FISH-inspired methods that leverage toehold probes to detect single nucleotide variants (SNVs) in RNA, including the first demonstration of visualizing A-to-I editing status of specific transcripts using inosine fluorescence *in situ* hybridization (inoFISH), thus providing insights into the link between editing patterns and localization.^32,33^ Approaches have also been designed to detect cytidine-to-uridine (C-to-U) editing of RNAs of interest in cells using forced intercalation (FIT) probes.^34,35^ A generalizable method for targeting polyadenylated RNAs coined click-encoded rolling FISH (ClickerFISH) was also developed and allowed the visualization of higher-order structures at the subcellular level with cell-to-cell variation.^36^ Most recently, deamination adjacent to RNA modification targets FISH (DART-FISH) was developed to enable *in situ* detection of individual m^6^A-modified and unmodified transcripts and determined that m^6^A alone is not sufficient to localize mRNA to stress granules.^37^ Together, these select examples highlight the importance of novel techniques to reveal the impact RNA modifications play intracellularly. Despite these advances that target specific transcripts, the need remains for a generalizable approach for quantifying and mapping the localization of inosine-containing RNAs across the transcriptome.^33^ Antibodies could prove advantageous toward this goal, yet efforts to generate an anti-inosine antibody have fallen short, with the resulting affinity reagents lacking the affinity and reproducibility needed for rigorous analytical methods (Figure S1).^38^ Taken together, a method for detecting and quantifying A-to-I edited RNA *in situ* remains an unmet challenge.

To address this need, we report herein an endonuclease V immunostaining assay (EndoVIA), which enables visualization of inosine-containing transcripts in cells using endonuclease V (EndoV, Figure 2b). EndoV is a magnesium-dependent ribonuclease that cleaves inosine-containing nucleic acid substrates; however, its activity can be controlled by replacing its natural magnesium cofactor with calcium such that EndoV binds inosine with high specificity without initiating cleavage.^39−42^ Our group has leveraged this binding event to ultimately repurpose EndoV as an “anti-inosine antibody” for enriching and quantifying edited RNA *in vitro*, which has in turn led to improved mapping of editing sites via RNA-seq as well as an ELISA-inspired microplate-based assay for quantifying global editing levels.^43,44^ While our group’s previous efforts have leveraged EndoV to accurately quantify A-to-I editing, these methods still require RNA extraction and pooling of RNA from a population of cells. These steps effectively erase information regarding the spatial distribution of edited RNA *in situ* as well as variation in editing signatures between cells. Seeking to overcome the limitations of these *in vitro* approaches, we recognized that the inosine binding capabilities of EndoV could be harnessed for detecting A-to-I edited transcripts *in situ* to retain RNA localization and cellular heterogeneity by recapitulating the principles of immunofluorescence (Figure 2c). After optimization of our immunostaining protocol, we validated our EndoVIA workflow using cells with varying levels of A-to-I editing. We then demonstrated that our workflow could be used to detect both elevated levels of A-to-I editing in a hyper-editing cancerous cell line and reduced editing levels in a hypo-editing cancerous cell line. Finally, we used total internal reflection fluorescence (TIRF) illumination coupled with super-resolution microscopy to detect edited RNA at the single-molecule level. Excitingly, we observe distinct subcellular localization patterns for edited RNA in both healthy and diseased cell line models. Together, these results demonstrate that EndoVIA can provide spatial visualization of A-to-I editing, enable quantification of total cellular inosine content at the single-cell level, and be used to map subcellular localization of edited transcripts. This in turn provides a powerful tool for acquiring previously unattainable insights into the localization of A-to-I editing and its role in cellular processes and disease progression.

## Results

### Development of EndoVIA for *in Situ* Imaging of A-to-I Edited Transcripts

Seeking to advance our use of EndoV as an “anti-inosine antibody” and develop a method for detecting edited RNA *in situ*, we turned to immunofluorescence for inspiration. Immunofluorescence is a technique used to image cellular components by directly or indirectly labeling targets of interest with fluorophore-tagged antibodies.^45^ The immunofluorescence staining process involves cellular fixation, permeabilization, blocking, and staining with a primary antibody specific for the target, followed by a secondary antibody conjugated to a fluorophore.^45^ Using this basic protocol for immunostaining as a scaffold, we integrated essential steps necessary for imaging inosine-containing RNA with EndoV and optimized this workflow in a simple model system, human embryonic kidney 293T (HEK293T) cells.

Our first goal was to strategically fix cells in a way that maximizes staining specificity by preserving RNA integrity and eliminating cellular components that could lead to potential off-target binding. Although widespread and effective, formaldehyde cross-links not only large RNAs such as rRNA and mRNA, but also small RNAs including tRNA that are edited within the anticodon loop by adenosine deaminases acting on tRNA (ADATs).^46,47^ While essential for cell viability, tRNA editing is unrelated to ADAR-mediated A-to-I editing, and tRNA is at least 100 times more abundant than mRNA in mammalian cells.^48^ Therefore, a significant concern was that fixation of tRNAs could mask our ability to visualize ADAR-edited targets. To circumvent this issue, we chose to fix cells using methanol (MeOH), another commonly used fixative reagent that retains large RNAs and allows small RNAs, like tRNA, to be washed away.^49,50^ To confirm that tRNA is indeed removed upon MeOH fixation, we performed tRNA FISH and observed that the presence of tRNA dramatically decreased after fixation and washing (Figure S2). In comparison to formaldehyde-fixed cells, methanol fixation decreased the presence of tRNA by ∼72% in two different cell lines (Figure S3, Figure S4).

In addition to the fixation method, we also explored additional workflow steps to achieve optimal EndoV binding of the target edited RNAs. Our group has shown that the commercially available, recombinant *Escherichia coli* EndoV binds edited single-stranded RNA (ssRNA) with a higher affinity than edited dsRNA.^43^ ADAR primarily targets RNA within double-stranded regions, and thus an RNA denaturation step is required to maximize EndoV binding to ADAR1-edited sites.^1^ We were inspired by our previous use of glyoxal to denature RNA secondary structure while preserving EndoV binding capability *in vitro* and hypothesized that we could recapitulate this approach in cells.^43^ Glyoxal is a reversible, chemical denaturant that forms bis-hemiaminal adducts with guanine, adenine, and cytosine nucleobases, thus disrupting Watson–Crick–Franklin base pairing.^43,44,51^ Importantly, glyoxal is unreactive toward inosine, as inosine differs from guanine by the lack of an exocyclic amine that is critical for glyoxal adduct formation. Following methanol fixation and glyoxal treatment, cells were permeabilized with detergent and blocked with bovine serum albumin (BSA) to prevent nonspecific binding. To achieve staining, cells were then incubated sequentially with EndoV, primary antibody, and fluorophore-labeled secondary antibody, all of which were diluted in a calcium-containing blocking buffer to support EndoV binding (Figure S5).

### Optimization of EndoVIA

Our workflow as described is representative of typical immunofluorescence experiments, with some modifications to support use of EndoV as an “anti-inosine antibody”. To confirm that our modified workflow was suitable for immunofluorescence and that the modifications made to enable use of EndoV would not alter cellular morphology or interfere with imaging, we first sought to detect and image well-known and characterized cellular components using commercially available antibodies. We chose to stain β-actin, a highly abundant structure in the cytoskeleton, and nucleoporin 153 (Nup153), a less abundant protein located in nuclear pore complexes.^52−55^ Images for both proteins were representative of previous reports, exhibiting sufficient signal and minimal background (Figure S6a and S6b).

Given that EndoV is fused to MBP, we considered the possibility that MBP could potentially engage in nonspecific binding and give rise to unwanted background signal. To assess nonspecific binding of the fusion protein, we stained HEK293T cells initially with a 1:1000 dilution EndoV-MBP fusion or *Escherichia coli* MBP using our previously outlined staining workflow and imaged using wide-field microscopy (Figure S7). Encouragingly, fluorescence was only observed for cells treated with EndoV-MBP, and cells treated with MBP only were comparable to negative controls. These results suggest the observed fluorescence is derived from the binding of EndoV to edited RNAs.

Detecting the maximum number of edited sites requires the complete denaturation of RNA secondary structure in order to grant EndoV access to these regions. To find the optimal glyoxal concentration for achieving this outcome, we stained *GAPDH* mRNA using FISH in HEK293T cells at varying concentrations of glyoxal (Figure S8). As RNA is increasingly glyoxylated, FISH probes are no longer able to hybridize to their targets, resulting in a decrease in fluorescence. We also stained β-actin in parallel under each glyoxal concentration to monitor cellular morphology (Figure S9). Taken together, we determined that 12% glyoxal was sufficient to denature RNA secondary structure without impacting cellular morphology.

Immunofluorescence protocols often require antibody optimization to minimize background and achieve maximum signal-to-noise ratio and sensitivity. To meet this need, we systematically screened EndoV concentrations to identify conditions where EndoV would stain the maximum number of targets while avoiding nonspecific binding of MBP. HEK293T cells were cultured and stained with increasing amounts of EndoV or MBP ranging from 1:4000 to 1:25, along with the appropriate controls (Figure S10). After quantifying the fluorescence, we determined that 1:50 EndoV was sufficient to achieve saturation while mitigating nonspecific binding of MBP. To confirm antibody specificity, we also stained HEK293T cells with the same workflow but omitting EndoV and observed no detectable fluorescence from control wells treated with only primary and secondary antibodies (Figure S11). For further rigor, we also hypothesized that removing calcium, which is essential for EndoV binding, would greatly reduce the fluorescence of inosine-containing RNA. To test this, we treated cells with EDTA following EndoVIA and found that fluorescence significantly decreased, further supporting that the signal observed could be attributed to EndoV binding to edited RNA (Figure S12).

### Validation of EndoVIA

**Figure 3:**
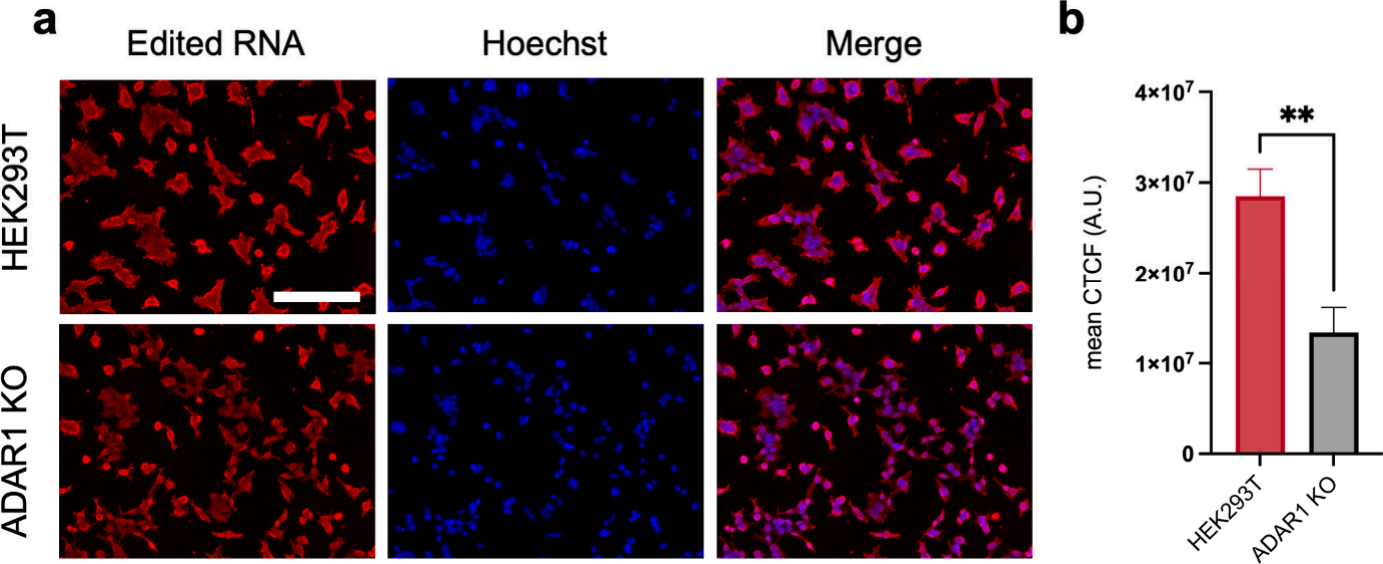
Staining edited RNA in cells with varying levels of A-to-I editing. (a) Fixed WT and ADAR1 KO HEK293T cells were immunostained using the optimized EndoVIA workflow for edited RNA (red) and cell nuclei (blue). (b) Quantification of mean corrected total cellular fluorescence (CTCF) in (a). Data in (a) and (b) are representative of three independent experiments; *n* = 3 wells from a 96-well plate. Scale bar, 200 μm. Data are shown as mean ± s.d. in arbitrary units (A.U.). Statistical significance was determined by unpaired *t*-test; **P < 0.01.

Having optimized our immunofluorescence protocol, we next sought to validate the ability of EndoVIA to detect biologically relevant differences in inosine by staining cells having varying levels of A-to-I editing. We predicted that if our workflow was targeting edited RNA as expected, then staining cells with little to no editing would lead to less fluorescence signal than staining of wild-type cells. To test our hypothesis, we chose to stain a HEK293T cell line with the ADAR1 gene knocked out via CRISPR-Cas9 genome editing.^9^ We first immunostained ADAR1 in both the WT and KO cell lines and confirmed that indeed ADAR1 was absent from the KO cell line (Figure S13). Using our optimized workflow, we then immunostained inosine-containing RNA in both wild-type and ADAR1 KO HEK293T cells and quantified the fluorescence (Figure 3a and 3b). Image analysis revealed that WT HEK293T cells show ∼2-fold greater fluorescence than ADAR1 KO cells, strongly suggesting that edited RNA is responsible for the staining.

While we were encouraged by this significant difference, we were not entirely surprised by the remaining fluorescence from the ADAR1 KO cells. Other enzymes that are large contributors of cellular inosine, such as ADAR2 and ADAT, are still present and may contribute to editing. We turned to RNA-seq and the Alu editing index (AEI) to compare the WT HEK293T and ADAR1 KO cells. The AEI was developed by Roth and co-workers and is a power computational tool that maps editing in Alu repeats across large data sets generated from RNA sequencing and has become widely accepted as the gold standard for quantifying ADAR editing activity.^56^ Briefly, the AEI value is generated from RNA-seq data by identifying sites that are called guanine in RNA but adenosine in DNA and then calculating the ratio of these edited sites to the total coverage of adenosines. We determined that the AEI values for the WT HEK293T and ADAR1 KO cells were 1.4 and 0.2, respectively (Figure S14). These results suggest that there is some remaining edited tRNA or other unwanted species giving rise to background signal. While methanol fixation removes the majority of tRNA, it is important to note that residual tRNA likely contributes to some of the observed fluorescence (Figure S4). Although the observed background signal was not ideal, we recognized that this could be especially noticeable in these very low-editing cells, whereas it might not be as problematic in other cell lines, and thus we carried forward. Encouragingly, our results described below indicate that this background signal appears to be nominal in the context of cells having more typical editing levels.

**Figure 4:**
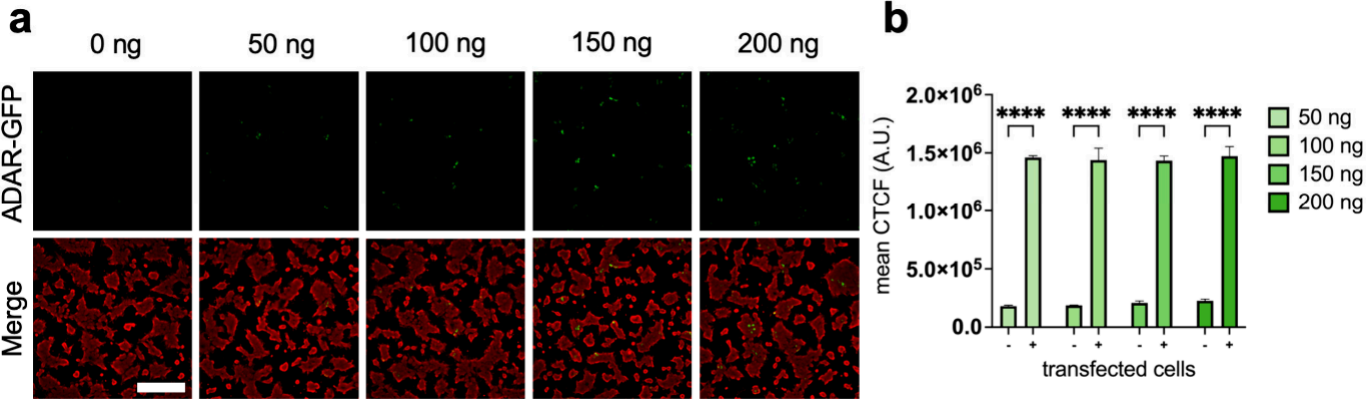
Detecting an increase in A-to-I editing in HEK293T cells. (a) HEK293T cells were transfected with increasing amounts of GFP-tagged ADAR1 p110 plasmid (0–200 ng, green), fixed, and stained for edited RNA (red) using EndoVIA. (b) Quantification of mean corrected total cellular fluorescence (CTCF) of edited RNA in ADAR-GFP positive (+) and negative (−) cells in (a). Data in (a) and (b) are representative of three independent experiments; *n* = 3 wells from a 96-well plate. Scale bar, 200 μm. Data are shown as mean ± s.d. in arbitrary units (A.U.). Statistical significance was determined by unpaired *t*-test; ****P < 0.0001.

We were curious to explore whether we could detect an increase in A-to-I editing as well. Immortalized cell lines have low editing activity in comparison to human primary cells. Specifically, HEK293T cells have low editing levels, making them an ideal cell line to stimulate ADAR1 upregulation.^57^ To explore this hypothesis, we transfected HEK293T cells with increasing amounts of a GFP-tagged ADAR1 p110 overexpression vector (pCMV) plasmid and stained accordingly (Figure 4a). We quantified the fluorescence of edited RNA in ADAR-GFP positive cells and determined that across all plasmid concentrations, cells expressing ADAR1-GFP displayed significantly higher immunostaining signal in comparison to cells that did not express ADAR1-GFP (Figure 4b). We observed the same trend in GFP-tagged ADAR1 p150 transfected cells as well (Figure S15). To confirm that changes in fluorescence were due to ADAR transfection, we also transfected HEK293T cells with increasing amounts of coilin-GFP, another nuclear protein. We found that there was no change in edited RNA fluorescence (Figure S16).

### Detecting A-to-I Editing in Cancer

Dysregulated A-to-I editing has been linked to various cancer types, and while many cancer types present a hyper-editing signature, some also display hypo-editing.^58^ Most notably, hyper-editing is evident in thyroid, head, neck, lung, and breast cancers, whereas hypo-editing is observed in metastatic melanoma, invasive breast cancer, and renal cancer.^20,59−61^ As a result, A-to-I editing is rapidly emerging as not only a biomarker for diagnosing and studying cancers but also a potential therapeutic target. We recognized that EndoVIA could prove to be particularly powerful in this context, as it could enable rapid quantification of editing levels across multiple samples and with the ability to observe cell-to-cell heterogeneity. Additionally, we envisioned that the ability to discriminate between inosine levels in healthy vs cancerous cells could serve as a foundation for the development of phenotypic screening assays for ADAR inhibition.

**Figure 5:**
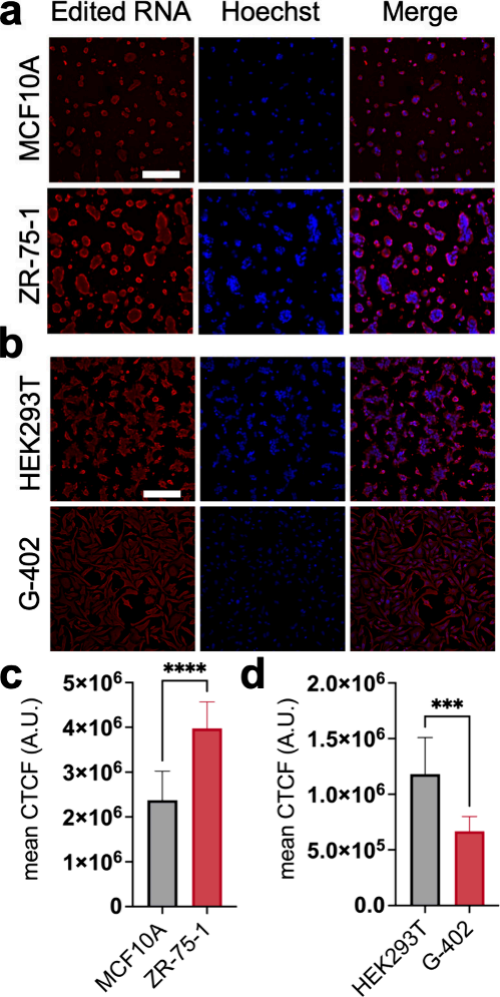
Identifying hyper- and hypo-editing in cancerous breast and kidney cells. (a) MCF10A cells (nonmalignant) and ZR-75-1 cells (malignant) and (b) HEK293T cells (nonmalignant) and G-402 cells (malignant) were fixed and stained for edited RNA (red) and cell nuclei (blue) using EndoVIA. (c) Quantification of mean corrected total cellular fluorescence (CTCF) in (a). (d) Quantification of mean CTCF in (b). Data in (a–d) are representative of three independent experiments; *n* = 9 wells from a 96-well plate. Scale bar, 200 μm. Data are shown as mean ± s.d. in arbitrary units (A.U.). Statistical significance was determined by unpaired *t*-test; ***P < 0.001, ****P < 0.0001.

Fortuitously, Schaffer et al. recognized the importance of having proper cellular models that reflect the A-to-I editing levels of a given tissue condition or disease signature, and using the AEI computational tool, they determined the AEI values for more than 1,000 human cell line types.^62^ This provided a wealth of validated cell lines for us to choose from in exploring the ability of EndoVIA to detect cancer-related changes in inosine levels. Drawing inspiration from the Cell Line A-to-I Editing Catalogue, we first chose to test our workflow against ZR-75-1 and MCF10A cell lines. ZR-75-1 cells are a malignant breast cell line that displays hyper-editing signatures with an AEI value of 2.1, whereas the MCF10A breast cell line is a nonmalignant control that has an AEI value of 1.4 (Figure 5a). Both cell lines were subjected to our EndoVIA workflow, and we were very excited to see that the ZR-75-1 cell line did indeed show higher fluorescence than the MCF10A cells (Figure 5c). Even more encouraging, the ratio between the immunofluorescence signal for the two cell lines was comparable to that of the reported AEI values. This demonstrates that EndoVIA is able to detect a cancer-related increase in RNA editing without the need for sequencing, which dramatically increases potential throughput.

Having established the ability of EndoVIA to detect cancer-related hyper-editing, we were also curious to explore whether we could detect cancer-related hypo-editing. Kidney cancers such as kidney chromophobe (KICH) and kidney renal papillary cell carcinoma (KIRP) in particular have been identified to display hypo-editing.^63^ Therefore, we selected the G-402 kidney cell line, characterized with renal leiomyoblastoma and an AEI value of 0.8. We also stained HEK293T cells that have an AEI value of 1.1 in parallel as a nonmalignant counterpart (Figure 5b). As anticipated, we were able to detect a decrease in fluorescence in the G-402 cell line (Figure 5d). We also confirmed that there were comparable amounts of mRNA across each pair of healthy and diseased cell lines, strongly suggesting that differences in fluorescence can be attributed to changes in A-to-I editing (Figure S17).

Having demonstrated that EndoVIA can detect global changes in inosine levels between healthy and cancerous cells, we sought to also capture nuanced shifts in cell-to-cell variation, as cellular heterogeneity is a hallmark of cancer. Using the CTCF values of individual cells across all four cell lines, we employed kernel density estimation to unveil the distribution of fluorescence within each cell population.^64^ There was a notable difference in the distribution patterns that emerged between healthy and diseased cells. Particularly striking was the comparison between HEK2393T and G-402 cells, where we observed a distinct migration of the density peak. In HEK293T cells, a higher density of cells exhibited elevated levels of A-to-I editing, while in G-402 cells the density shifted toward lower CTCF values, signaling an increase in cells displaying reduced levels of editing (Figure S18a). A similar trend was also observed in MCF10A and ZR-75-1 cells, where higher A-to-I editing levels in ZR-75-1 cells displayed a shift in cell density toward higher CTCF values (Figure S18b). These findings aligned with our expectations, reinforcing that EndoVIA is capable of not only detecting widespread changes in A-to-I editing but also uncovering nuances in cellular heterogeneity.

EndoVIA serves as the first method capable of detecting inosine-containing transcripts *in situ*; thus we were curious about its performance against traditional approaches for characterizing A-to-I editing in cells, namely, dsRNA antibodies. Given that dsRNA is the primary target of ADAR, the J2 and K1 antibodies have been routinely employed for detecting and enriching ADAR substrates. To put these antibodies to the test, we chose to stain dsRNA with J2 and K1 in HEK293T and G-402 cells and compare to EndoVIA that was completed in parallel (Figure S19). The localization patterns for all three treatments varied across both HEK293T and G-402 cells. Notably, the dsRNA antibodies exhibited a surprising absence of fluorescence in the nucleus, despite this being ADAR’s primary residence for RNA editing. K1, specifically in HEK293T cells, showed localization patterns most comparable to EndoV, demonstrating increased fluorescence near the cell membrane. These observable differences across treatments underscore the unknown binding preferences of J2 and K1 in the context of A-to-I editing status. In comparison, EndoVIA offers a higher degree of confidence in staining edited RNA, as it directly detects inosine, providing a higher level of detail and reliable staining approach.

### Detecting Subcellular Localization of Inosine-Containing Transcripts

In addition to the ability to rapidly detect global inosine levels in cell samples, we envisioned that EndoVIA might also enable imaging of the subcellular localization of edited RNAs. The majority of A-to-I editing occurs within the 3′ UTRs of mRNA, which heavily influences RNA localization. The loss of an edited 3′ UTR has been shown to completely alter the destination of a given RNA.^22−25^ Despite this evident link between A-to-I editing and RNA localization, many unanswered questions remain due to the lack of available methods. As an example of this, the images of HEK293T cells presented in the figures show observable disparities in fluorescence signal, with signal seeming to be concentrated near the cellular membrane. In order to more rigorously study these localization patterns and achieve greater resolution, we hypothesized that TIRF illumination coupled with super-resolution microscopy would yield high-quality, nanometric spatial insight from EndoVIA. Thus, we performed the immunostaining on both HEK293T and G-402 cell lines using the EndoVIA protocol.

**Figure 6:**
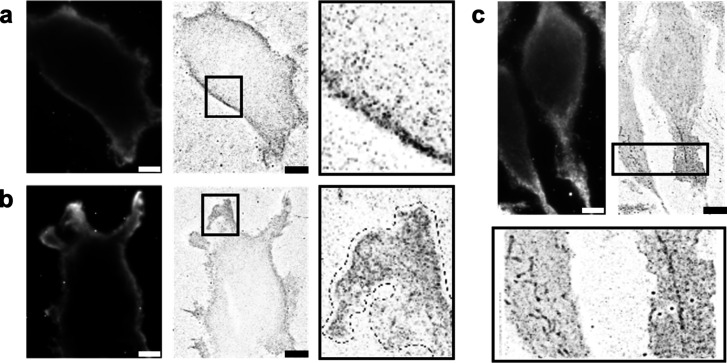
Super-resolution microscopy of edited RNA using EndoVIA. TIRF (dark images) and TIRF-dSTORM images (light images, whole cell and close-up view of boxed regions, respectively) from (a) HEK293T cells and (b) adhesion site of HEK293T cells and (c) G-402 cells labeled with the described immunostaining workflow. Data in (a–c) are representative of three independent experiments; *n* = 3 coverslips. Scale bar, 10 μm.

Imaging of HEK293T cells using dSTORM in TIRF illumination enabled us to observe single EndoV binding events (presumably from individual editing sites) with large populations near the membrane (Figure 6a) and at the adhesion site (Figure 6b). Interestingly, while we observe edited RNAs in both subcellular locations, the edited RNA is concentrated at the cellular membrane in contrast to displaying a more even distribution throughout the adhesion sight. Interestingly, previous reports have determined that cellular adhesion and motility are partly regulated through RNA localization and translation at focal adhesions.^65,66^ Encouragingly, when cells were stained omitting EndoV and imaged using dSTORM in TIRF illumination, there was no detectable fluorescence and thus was consistent with previous experiments (Figure S20). Together, these findings offer new potential insights into the downstream functions of editing, as well as mark the first demonstration of a method that provides spatial distribution of A-to-I editing across the cell at the single-molecule level.

dSTORM TIRF imaging of cancerous G-402 cells revealed similar localization of edited RNAs at the membrane, though less pronounced than in the HEK293T cells. G-402 cells also appear to display more even distribution of edited transcripts throughout the cytoplasm. Interestingly, upon closer inspection, super-resolution imaging of the G-402 cells reveals some clustering in the cytoplasm that may represent subcellular structures for organizing edited RNAs that are not present in HEK293T cells and not visible using standard TIRF or confocal imaging (Figure 6c). Despite these differences in the localization of edited RNA, ADAR1 localization in both HEK293T cells and G-402 cells remains in the nucleus, hinting at potential underlying mechanisms dictating the localization of edited transcripts that have yet to be discovered (Figure S21). These results not only underscore the power of EndoVIA to lead to new biological discoveries and knowledge but also establish its compatibility with advanced imaging modalities.

These results represent the first example of transcriptome-wide visualization of edited RNAs with nanoscale resolution in the cellular environment. We do note that some residual tRNA can be present after the fixation steps and may lead to background signal. However, tRNA FISH in the HEK293T cells reveals that this remaining tRNA is primarily sequestered to nuclear foci (Figure S3), and we do not observe a strong corresponding EndoV signal in our single-molecule EndoVIA experiments. This suggests that the interference of residual tRNA is minimal, though out of an abundance of caution we do recommend that tRNA FISH be included as a control experiment when using EndoVIA to map subcellular localization of edited RNAs. Together, these experiments open up a wide range of potential explorations that will reveal new links between A-to-I editing and RNA localization and in turn provide novel insights into the role of this important process in development and disease.

## Discussion

A-to-I editing is the most widespread post-transcriptional modification and is essential for multiple biological processes. One way that editing impacts cellular pathways is thought to be through directing the localization of RNAs to subcellular compartments by editing within the 3′ UTR of mRNAs. Although there are a few demonstrations that nuclear retention of specific mRNAs is a result of A-to-I editing, the broader impact of editing on RNA localization is underexplored due to a lack of methods for imaging inosine-containing RNAs in the cellular context. In parallel, dysregulation of A-to-I editing is intricately linked to neurological disorders, autoimmune diseases, and multiple cancers, highlighting its critical role in disease pathogenesis. However, the tremendous potential of editing to serve as a biomarker or therapeutic target is limited by the lack of methods for observing editing directly *in situ*. Specifically, the current gold standard technique of high-throughput RNA sequencing requires RNA extraction and pooling of RNA from multiple cells in a sample. This essentially erases information relating to cell-to-cell variation of global inosine levels and precludes studying the specific subcellular localization of edited RNAs. Moreover, RNA-seq remains limited in throughput, making it poorly suited for high-throughput drug screening campaigns.

Herein, we introduce EndoVIA as the first approach for imaging and quantifying the full breadth of inosine-containing transcripts in cells. Key to the development of this technique is our repurposing of EndoV to act as an “anti-inosine antibody”, which in turn enables us to develop a protocol analogous to immunofluorescence staining that is aimed at detecting edited RNAs *in situ*. We have validated our approach using cells having varying editing levels and demonstrate the ability to detect biologically relevant differences in inosine between healthy and cancerous cell lines. We also show that EndoVIA is compatible with super-resolution microscopy techniques to image edited RNAs at nanoscale resolution and provide previously unattainable insight into the subcellular localization and organization of edited transcripts.

We envision that the availability of EndoVIA will open up numerous avenues for studying RNA editing and harnessing this important process for diagnostics and therapeutics. For example, efforts are underway in our laboratory to further elaborate and refine EndoVIA to enable high-throughput phenotypic screening for the discovery of novel small molecules to regulate A-to-I editing. Additionally, the ability to image the subcellular localization of edited RNAs can be directed toward probing a wide range of biological questions regarding how this localization is impacted by cell type, disease state, and external stimuli. Thus, this first-in-class approach for imaging A-to-I editing at the cellular level is expected to not only advance research in our own laboratory but also empower other groups studying A-to-I editing and in turn advance both the basic science and translational potential of this important biological process.

## Supporting Information Available

Experimental methods, sources of materials, and nucleic acid sequences, as well as Figures S1 and S21 (PDF)
Transparent Peer Review report available (PDF)
