# Supplementary material for: Spatial Visualization of A-to-I Editing in Cells Using Endonuclease V Immunostaining Assay (EndoVIA)
Source: ACS Cent Sci. 2024 Jul 8;10(7):1396–405. doi: 10.1021/acscentsci.4c00444 (PMC11273454; doi:10.1021/acscentsci.4c00444)
Supplement: Supplementary file 1 — oc4c00444_si_001.pdf [file oc4c00444_si_001.pdf]

Spatial Visualization of A-to-I Editing in Cells using Endonuclease V Immunostaining Assay (EndoVIA)

Authors: Alexandria L. Quillin[1], Benoît Arnould[1], Steve D. Knutson[2], and Jennifer M. Heemstra[1*]

[*]To whom correspondence should be addressed: heemstra@wustl.edu

Affiliations: [1]Department of Chemistry, Washington University in St. Louis, MO 63130, USA. [2]Merck Center for Catalysis, Princeton University, Princeton, New Jersey 08544, USA. Department of Chemistry, Princeton University, Princeton, New Jersey 08544, USA.

Supplemental Information

Table of Contents

Materials and Methods S2-S7
Table S1. Synthetic oligoribonucleotides used in this study S8
Table S2. tRNA FISH Probes S8
Figure S1. Binding curves of anti-inosine antibodies S9
Figure S2. Fluorescence *in situ* hybridization (FISH) of tRNA using different fixatives S10
Figure S3. Detection of tRNA in formaldehyde-fixed and methanol-fixed cells S11
Figure S4. Quantification of residual tRNA in methanol-fixed cells S12
Figure S5. Summary of EndoVIA workflow S13
Figure S6. Detection of Nup153 and β-actin using modified immunofluorescence workflow S14
Figure S7. Non-specific binding of MBP of the EndoV-MBP fusion protein S15
Figure S8. Optimizing glyoxal concentration using *GAPDH* fluorescence *in situ* hybridization S16
Figure S9. Cellular morphology of cells treated with varying glyoxal concentrations using β-actin S17
Figure S10. Optimizing EndoV concentration S18
Figure S11. Antibody negative control for confocal imaging S19
Figure S12. EndoV stained cells treated with EDTA S20
Figure S13. Immunostaining ADAR1 S21
Figure S14. AEI Values of WT and ADAR1 KO HEK293T cells S22
Figure S15. Detecting A-to-I editing in HEK293T cells transfected with ADAR1-p150-GFP S23
Figure S16. Quantifying A-to-I editing in HEK293T cells transfected with coilin-GFP S24
Figure S17. Quantifying mRNA in non-malignant and malignant cell lines S25
Figure S18. Cellular heterogeneity in non-malignant and malignant cell lines S26
Figure S19. Immunostaining dsRNA and edited RNA in healthy and diseased cells S27
Figure S20. Antibody negative control for dSTORM in TIRF illumination imaging S28
Figure S21. Immunostaining ADAR1 in HEK293T and G-402 cells S29

Materials and Methods

Safety Statement

No unexpected or unusually high safety hazards were encountered in this study.

Materials

Methanol, 4% paraformaldehyde, ethanol, 40% glyoxal, acetic acid, Triton X-100, Tween 20, tris hydrochloride, calcium chloride, sodium chloride, cholera toxin, TE buffer, and EDTA were purchased from Sigma Aldrich. Nuclease-free water, Hoechst 33342, and 6-well plates were purchased from Thermo Fisher Scientific.

RNA Oligoribonucleotides

All oligoribonucleotides used in this study were custom designed and purchased from Integrated DNA Technologies (IDT). Complete sequences can be found in Table S1.

Cell Culture and Transfection

The G-402 cell line (ATCC) was cultured in McCoy's 5a Medium Modified (ATCC) supplemented with 10% fetal bovine serum (Gibco) and 1% penicillin-streptomycin (Gibco). The HEK293T cell line (ATCC) and the HEK293T ADAR1 KO cell line (gifted by Dr. Charles Rice) was cultured in Dulbecco's Modified Eagle's Medium (Gibco) supplemented with 10% fetal bovine serum and 1% penicillin-streptomycin. The MCF10A cell line (ATCC) was cultured in MEBM Basal Medium (Lonza) supplemented with the included additives, excluding GA-1000, and 100 ng mL^{-1} cholera toxin. The ZR-75-1 cell line (ATCC) was cultured in RPMI-1640 Medium (ATCC) supplemented with 10% fetal bovine serum and 1% penicillin-streptomycin. All cell lines were cultured at 37°C in a humidified incubator with 5% CO_2. For FISH and immunofluorescence experiments, black 96-well plates (Cellvis) were coated in poly-D-lysine (Gibco) following the manufacturer's protocol. Cells were seeded at a density of 10,000 cells per well followed by a 48-hour incubation at 37°C in a humidified incubator with 5% CO_2. For transfection, cells were seeded as previously described and 24 hours post seeding (~70% confluent), cells were transfected with increasing amounts of ADAR1-GFP plasmid (p110 and p150, Addgene) or coilin-GFP plasmid (Addgene) using Opti-MEM Reduced Serum Medium (Gibco) and lipofectamine 3000 (Invitrogen) following the manufacturer's protocol. Cells were then incubated for 48 hours at 37°C in a humidified incubator with 5% CO_2 before completing subsequent immunostaining.

tRNA and GAPDH Fluorescence *In Situ* Hybridization (FISH)

For tRNA FISH, cells were seeded as previously described. Wells were then fixed with ice cold 100% methanol or 4% paraformaldehyde. Methanol treated wells were incubated at 4°C for 20 minutes and paraformaldehyde treated wells were incubated at room temperature for 15 minutes. Fixative reagents were then removed, and wells were washed with 1X PBS (Invitrogen) for 1 hour at room temperature. Cells were permeabilized in 0.1% Triton X-100 in 1X PBS for 10 minutes. The Stellaris RNA FISH hybridization buffer, Wash Buffer A, and Wash Buffer B were prepared according to the manufacturer's instructions (Stellaris). The 1X PBS was then replaced with the prepared Stellaris RNA FISH Wash Buffer A and incubated at room temperature for 5 minutes. tRNA FISH probes (IDT, Table S1) were dissolved in TE buffer (10 mM tris hydrochloride,

1 mM EDTA, pH 8.0) at a concentration of 100 μM and then diluted in the prepared hybridization buffer to a concentration of 250 nM. Wash Buffer A was then replaced with the diluted probes, the plate was sealed to prevent evaporation, and incubated in the dark at 37°C overnight. The probes were removed and replaced with Wash Buffer A and incubated in the dark at 37°C for 30 minutes. Wash Buffer A was removed and replaced with Wash Buffer B and incubated at room temperature for 5 minutes. Wash Buffer B was exchanged for 1X PBS and cells were imaged. For GAPDH FISH, cells were seeded as previously described. Wells were then fixed with ice cold 100% methanol at 4°C for 20 minutes. The methanol was replaced with 1X PBS and washed at room temperature for 1 hour. Wells were treated with either 3%, 6%, 12%, or 30% glyoxal solution and incubated at 50°C for 1 hour. The glyoxal solutions were removed and wells were washed twice with 1X PBS. Cells were then permeabilized in 0.1% Triton X-100 in 1X PBS for 10 minutes. GAPDH FISH was completed by using Human GAPDH FISH Probes (Stellaris) and the previously described protocol for tRNA FISH. Cells were then imaged. FISH was completed under nuclease-free conditions.

Immunofluorescence

Nup153 and β-actin were immunstained to ensure that the EndoVIA workflow has the dynamic range needed to capture the signal associated with edited RNA. These control proteins were immunostained in in the following manner. Cells were cultured and seeded as previously described. Cells were then fixed in 100% ice cold methanol and incubated at 4°C for 20 minutes. The methanol was removed, and wells were washed twice with 1X PBS. Cells were next rehydrated with 1X PBS by incubating for 1 hour at room temperature with gentle shaking. A 4% glyoxal denaturing solution from Fu and Zhuang was prepared by combining the following in order: 1.974 mL nuclease-free water, 0.796 mL of 100% ethanol, 1.200 mL of glyoxal, and 0.030 mL acetic acid and incubated at 37°C for 1 hour.[1] Wells were then washed twice with 1X PBS. The 1X PBS was replaced with a 0.1% Triton X-100 in 1X PBS permeabilization solution and incubated at room temperature for 10 minutes followed by two washes in 1X PBS. Cells were then incubated in a blocking solution containing 3% bovine serum albumin (Gibco), 0.1% Triton X-100, and 0.1% Tween 20 in 1X PBS for 1 hour at room temperature. Wells were washed twice in 1X PBS and then incubated in 1:100 anti-Nup153 antibody (Abcam) or 1:1000 anti-β-actin antibody (Invitrogen) in blocking buffer for 1 hour at room temperature. The primary antibody solutions were removed, and wells were washed three times in 1X PBS. Finally, a staining solution comprised of 1:1000 Hoechst nuclear dye and 1:1000 goat anti-rabbit Alexa Fluor 647 (Invitrogen, Nup153) or 1:1000 goat anti-rabbit Alexa Fluor 488 (Invitrogen, β-actin) diluted in blocking buffer were added and incubated for 1 hour at room temperature in the dark. Wells were then washed in 1X PBS three times and imaged. As negative controls, cells were also stained by omitting the primary or secondary antibodies to assess nonspecific binding and autofluorescence. To assess the cellular morphology under varying glyoxal conditions, β-actin was stained in the following manner. Cells were cultured and seeded as previously described. Cells were then immunostained as previously described, with the exception that multiple glyoxal concentrations were tested (4%, 12%, 30%). ADAR1 and dsRNA were stained in the following manner. Cells were cultured and seeded as previously described. Cells were then fixed in 4% paraformaldehyde at room temperature for 15 minutes and then washed,

permeabilized, and blocked as previously described for Nup153 and β-actin. After washing with 1X PBS, cells were incubated in primary antibody solutions consisting of 1:25 anti-dsRNA J2 antibody (Sigma Aldrich), 1:25 anti-dsRNA K1 antibody (Nordic MUbio), or 1:400 anti-ADAR1 antibody (Atlas Antibodies) in blocking buffer and incubated for 1 hour at room temperature. The primary antibody solutions were removed, and wells were washed three times in 1X PBS. Finally, a staining solution comprised of 1:1000 Hoechst nuclear dye and 1:500 goat anti-mouse Alexa Fluor 647 (Invitrogen, J2 and K1) or 1:1000 goat anti-rabbit Alexa Fluor 647 (Invitrogen, ADAR1) diluted in blocking buffer were added and incubated for 1 hour at room temperature in the dark. Wells were then washed in 1X PBS three times and imaged. All immunostaining incubations were completed with 200 mL volumes and solutions were prepared fresh for each experiment. Immunofluorescence was completed under nuclease-free conditions.

EndoVIA

Cells were seeded as previously described. Cells were then fixed in 100% ice cold methanol and incubated at 4°C for 20 minutes. The methanol was removed, and wells were washed twice with 1X PBS. Cells were next rehydrated with 1X PBS by incubating for 1 hour at room temperature with gentle shaking. A 12% glyoxal denaturing solution inspired by Fu and Zhuang was prepared by combining the following in order: 1.974 mL nuclease-free water, 0.796 mL of 100% ethanol, 1.200 mL of glyoxal, and 0.030 mL acetic acid.[1] These volumes were scaled up or down as needed for the experiment. The 1X PBS was removed and the glyoxal denaturing solution was placed on cells and incubated at 50°C for 1 hour. Cells were then incubated with a 0.1% Triton X-100 in 1X PBS permeabilization solution at room temperature for 10 minutes followed by two washes in 1X PBS. Cells were then incubated in a blocking solution containing 3% bovine serum albumin, 0.1% Triton X-100, and 0.1% Tween 20 in 1X PBS for 1 hour at room temperature. Wells were then washed twice with 1X PBS. A calcium containing blocking buffer was prepared as follows: 3% bovine serum albumin and 1 mM calcium chloride in 1X PBS. Cells were incubated with 1:50 Endonuclease V (New England BioLabs) in the calcium containing blocking buffer, or the calcium containing blocking buffer alone as a negative control, at room temperature for 1 hour. A calcium containing wash buffer with 1 mM calcium chloride, 20 mM tris hydrochloride, and 150 mM sodium chloride was made and used for the remainder of the washing steps. Cells were then washed three times, incubating for 5 minutes each time. The commercially available EndoV is fused to a maltose-binding protein (MBP) tag that is commonly used for purification purposes. Thus, we chose to use an anti-MBP primary antibody that binds MBP of the EndoV-MBP fusion protein. Cells were then incubated for 1 hour at room temperature with a 1:1000 anti-MBP antibody (New England BioLabs) solution diluted in calcium containing blocking buffer. Cells were washed three times, with 5-minute incubation periods. Finally, a staining solution containing 1:1000 goat anti-mouse Alexa Flour 647 and 1:1000 Hoechst nuclear dye in calcium containing blocking buffer was incubated in the wells for 1 hour at room temperature in the dark. Cells were then washed three times for 5 minutes each time. Cells were then imaged. Because EndoV is used in a manner analogous to an antibody for inosine, optimizing concentration was thus imperative for developing a robust fluorescence-based assay that accurately detects edited substrates. The EndoV concentration used for the EndoVIA workflow was determined by following the previously

described protocol using a range of EndoV and MBP (Novus Biologicals) concentrations (1:4000-1:25). As a negative control, a set of cells were not treated with EndoV fusion or MBP but were still incubated with primary and secondary antibody.The calcium dependence of EndoVIA was confirmed by treating wells with 5 mM EDTA and incubating for 1 hour at room temperature following EndoVIA and then imaged. All immunostaining incubations were completed with 200 mL volumes and solutions were prepared fresh for each experiment. EndoVIA was performed under nuclease-free conditions.

mRNA Quantification
Cells were seeded in 6-well plates at a density of 300,000 cells per well and incubated at 37°C in a humidified incubator with 5% CO_2 for 24 hours before harvesting for RNA. Cells were lysed and mRNA was isolated and purified using the Magnetic mRNA Isolation Kit (New England BioLabs). Resulting purified mRNA was quantified using Nanodrop.

Microscopy and Image Analysis
Stained cells were imaged using a Nikon Spinning Disk for widefield microscopy with a 20x air objective and confocal microscopy with a 60x oil objective. Laser excitation at 405 nm was used to image Hoechst 33342; excitation at 640 nm was used to image Alexa Fluor 647; excitation at 488 nm was used to image ADAR-GFP; excitation at 560 nm was used to image Quasar570 labeled GAPDH FISH probes. Gain and exposure settings for each laser were optimized to achieve sufficient fluorescence and minimize oversaturation. The resulting images were analyzed to determine the fluorescence of each cell using FIJI. The area and integrated density were measured for each cell and then the Corrected Total Cellular Fluorescence was calculated as follows: $CTCF = integrated\ density - (cell\ area \times mean\ background\ fluorescence)$.

Alu Editing Index (AEI)
Cells were seeded in 6-well plates at a density of 300,000 cells per well and incubated at 37°C in a humidified incubator with 5% CO_2 for 48 hours. Cells were lysed and total RNA was isolated and purified using the Monarch Total RNA Miniprep Kit (New England BioLabs). This purified RNA was then used to prepare sequencing libraries with the Tru-Seq Stranded with RiboZero Gold (Human/Mouse/Rat) Kit. Standard 8-bp i5 and i7 Illumina index barcode and adapters were added to each library. Libraries were sequence using a NovaSeq X Plus 300 cycles (Illumina) to produce paired end 150-bp reads (approximately 30M reads per sample). Raw FASTQ files were trimmed using Trimmomatic1 with the parameter HEADCROP:3 to remove the first 3 bp.[2] FASTQC (https://www.bioinformatics.babraham.ac.uk/projects/fastqc/) was then used to check read quality (PHRED33) after data trimming. Reads were next aligned to the human reference genome hg38 using STAR 2.5.23 with the additional parameter --outFilterMatchNminOverLread 0.95 to detect A-to-I editing.[3] The resulting .bam files were sorted, and duplicates were removed using Samtools 1.35.[4] Finally, the RNA editing indexer package was used with the default settings to calculate the AEI for each sample.[5]

(1) Ye Fu, X. Z. m6A-Binding YTHDF Proteins Promote Stress Granule Formation. *Physiol. Behav.* **2016**, *176* (3), 139–148. https://doi.org/10.1038/s41589-020-0524-y.m.

(2) Bolger, A. M.; Lohse, M.; Usadel, B. Trimmomatic: A Flexible Trimmer for Illumina Sequence Data. *Bioinformatics* **2014**, *30* (15), 2114–2120. https://doi.org/10.1093/bioinformatics/btu170.
(3) Dobin, A.; Davis, C. A.; Schlesinger, F.; Drenkow, J.; Zaleski, C.; Jha, S.; Batut, P.; Chaisson, M.; Gingeras, T. R. STAR: Ultrafast Universal RNA-Seq Aligner. *Bioinformatics* **2013**, *29* (1), 15–21. https://doi.org/10.1093/bioinformatics/bts635.
(4) Li, H.; Handsaker, B.; Wysoker, A.; Fennell, T.; Ruan, J.; Homer, N.; Marth, G.; Abecasis, G.; Durbin, R. The Sequence Alignment/Map Format and SAMtools. *Bioinformatics* **2009**, *25* (16), 2078–2079. https://doi.org/10.1093/bioinformatics/btp352.
(5) Roth, S. H.; Levanon, E. Y.; Eisenberg, E. Genome-Wide Quantification of ADAR Adenosine-to-Inosine RNA Editing Activity. *Nat. Methods* **2019**, *16* (11), 1131–1138. https://doi.org/10.1038/s41592-019-0610-9.

Kernel Density Estimation
This analysis utilized Python with the pandas, seaborn, and matplotlib libraries to create Kernel Density Estimation (KDE) plots from the individual CTCF values of each cell. The CTCF values underwent preprocessing and were loaded into a pandas DataFrame. KDE plots were then generated using the seaborn library's kdeplot () function. The python code was written and executed using Visual Studio Code.

dSTORM super-resolution Setup and Imaging
dSTORM images were acquired on a home-built setup based on a Nikon Ti2 Eclipse microscope body operated with Micro-Manager 2.0. Excitation was performed by a 180mW 638nm laser (Cobolt 06-MLD, Coherent) to which the beam was circularly polarized (ThorLabs), expanded (GBE10-A , ThorLabs) and focused at the back focal plane of the objective (SR HP TIRF, 100X, 1.49NA, Nikon) by an achromatic lens (f=300mm, AC254-300-A, ThorLabs) mounted on a motorized translating stage (KMTS25E, ThorLabs) to achieve TIRF illumination. A filter cube (TRF89901v2, Chroma) containing excitation and emission filters ensure spectral filtering of both light paths. Emission was detected by a sCMOS camera (Orca Fusion, Hamamatsu) with 2x2 binning, resulting in effective pixel size of 130 nm. Prior to imaging, cells were treated with dSTORM imaging buffer (GLOX) consisting of 0.56 mg.mL-1 Glucose Oxidase (Sigma), 34 µg.mL-1 Catalase (Sigma), 50 mM Tris (pH 8.00), 10 mM NaCl, 10% glucose (w/v) and 143 mM β-mercaptothanol. For imaging, a stack of 20,000 images were taken per field at maximum laser power. The first 1,000 images were discarded and the remaining stacks were analyzed by ThunderSTORM imageJ plugin. Microscope's Perfect Focus System (PFS, Nikon) compensate for Z drift, while 100nm fiducial marker (TetraSpeck, Invitrogen) were added post-fixation to compensate for XY drift.

Glyoxal Treatment
200 pmol of RNA A or RNA I (Table S1) was added to 14.5 µL of 40% glyoxal, 50 µL of DMSO, and nuclease-free water to a final volume of 100 µL. Samples were reacted for 1 hour at 50 ˚C and ethanol precipitated.

Microscale Thermophoresis (MST)

For EndoV binding studies, varying amounts of EndoV (New England BioLabs) diluted in Diluent C (New England BioLabs) were combined with 0.4 pmol of the respective glyoxalated RNA A or RNA I targets in a final volume of 20 µL and incubated at room temperature for 30 minutes. Samples were then loaded into standard glass capillaries and MST was performed using a Nanotemper Monolith NT.115 Instrument. All measurements were analyzed using the Nano-Red filter with medium MST-Power and 100% excitation-power. Data were fitted using GraphPad Prism 8 to determine K_d values. For the anti-inosine antibody binding studies, varying amounts of each antibody were diluted in 50% glycerol 50% 1X PBS (MBL) or 1X PBS (Diagenode) and prepared and analyzed as previously described for EndoV.

Statistics

A minimum of three biological replicates were completed for each experiment. Statistical analyses were completed using GraphPad Prism 10 and all values and error bars indicate the mean ± s.d. unless otherwise noted. For comparison of two independent groups, an unpaired *t*-test was performed. For multiple comparisons, one-way ANOVA was performed.

Name	Sequence
RNA A	5' – CCC GCC AAC CCC GAG UUA GCG GGC /3Cy5Sp/ – 3'
RNA I	5' – CCC GCC AAC CCC GAG UUI GCG GGC /3Cy5Sp/ – 3'

Table S1. Synthetic oligoribonucleotides used in this study. The following synthetic oligoribonucleotides are labeled with Cy5 and were used for MST analysis.

tRNA Ser AGA	
Probe 1	5' – AA+C CA+C TC+G GC+C AC+G AC+T AC/3AlexF488N/ – 3'
Probe 2	5' – CGC +GGG +GAA +ACC +CCA +ATG +GAT +TTC /3AlexF88N/ –3'

Table S2. tRNA Fluorescence *in situ* hybridization (FISH) probes. The following locked nucleic acid (LNA) FISH probes were used to detect tRNA Ser AGA. LNA bases are denoted with (+) following the nucleobase and probes are labeled with Alexa Fluor 488 (3AlexF88N).

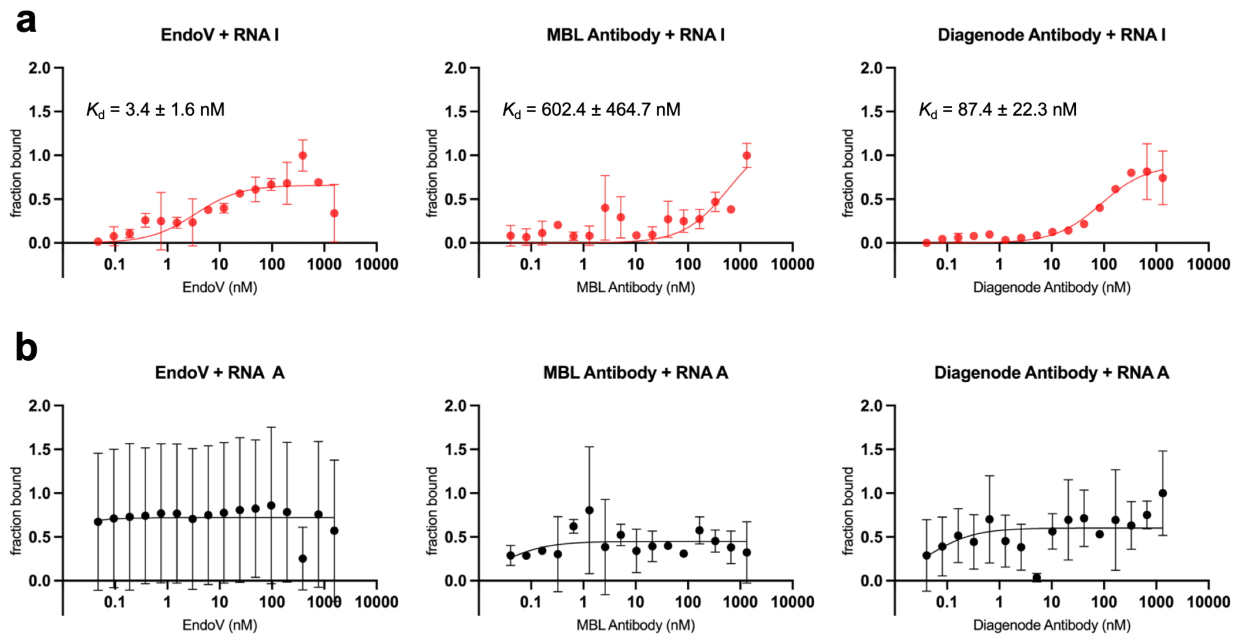

Figure S1. Binding curves of anti-inosine antibodies. Quantification of the MBL anti-inosine antibody and the Diagenode anti-inosine antibody binding affinity towards ssRNA I (red) and ssRNA A (black) using MST. Values represent mean with standard deviation, and K_d denotes mean with 95% confidence interval. Data are representative of three independent experiments; *n=3* individual trials.

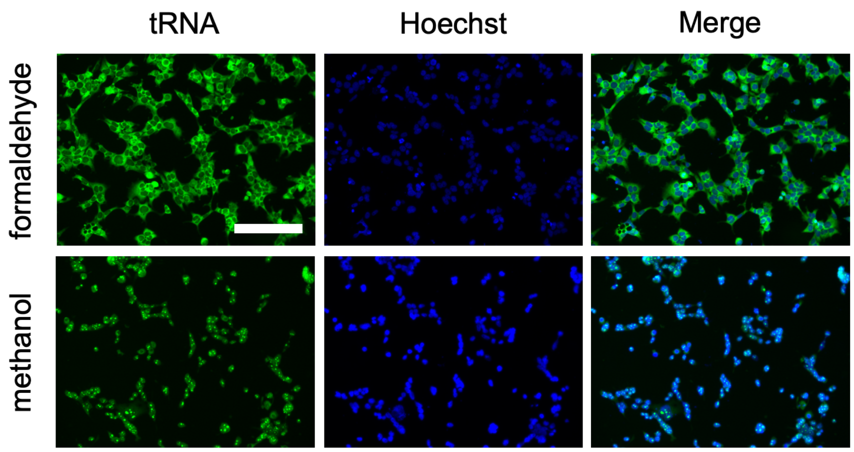

Figure S2. Fluorescence *in situ* hybridization (FISH) of tRNA using different fixatives. HEK293T cells were fixed with either formaldehyde or methanol and stained for tRNA (green) and cell nuclei (blue). Data are representative of three independent experiments; *n=3* wells from a 96-well plate. Scale bar, 200 μm.

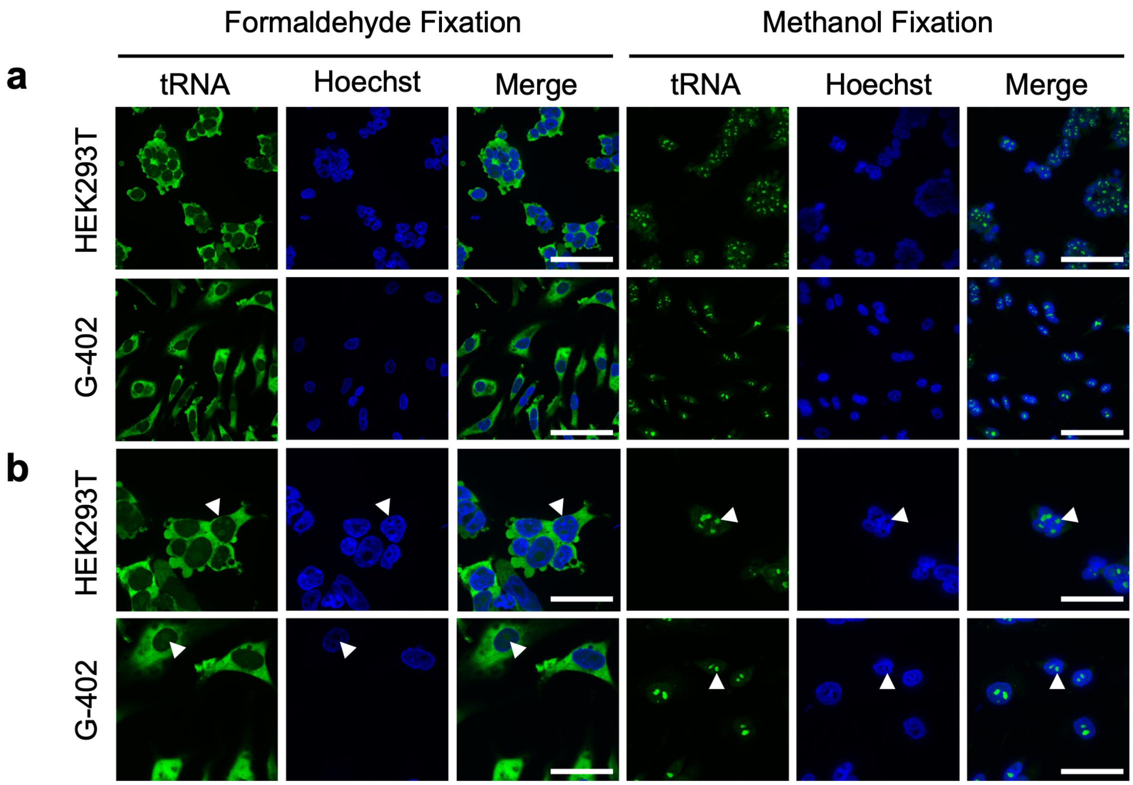

Figure S3. Detection of tRNA in formaldehyde-fixed and methanol-fixed cells. (a) HEK293T and G-402 cells were fixed with either formaldehyde or methanol and stained for tRNA (green) and cell nuclei (blue). (b) Close-up view of images in (a) with nuclear puncta identified using white triangles. Data are representative of three independent experiments; *n=3* wells from a 96-well plate. Scale bar, (a) 50 μm and (b) 25 μm.

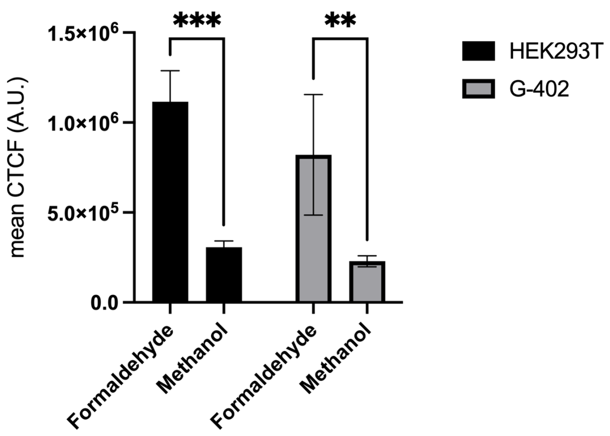

Figure S4. Quantification of residual tRNA in methanol-fixed cells. tRNA fluorescence in Figure S3 was quantified for HEK293T (black) and G-402 (gray) cells fixed with formaldehyde or methanol. Data are representative of three independent experiments; *n=3* wells from a 96-well plate. Data are shown as mean ± s.d. in arbitrary units (A.U.). Statistical significance was determined by unpaired *t*-test; $^{**}P < 0.01$, $^{***}P < 0.001$.

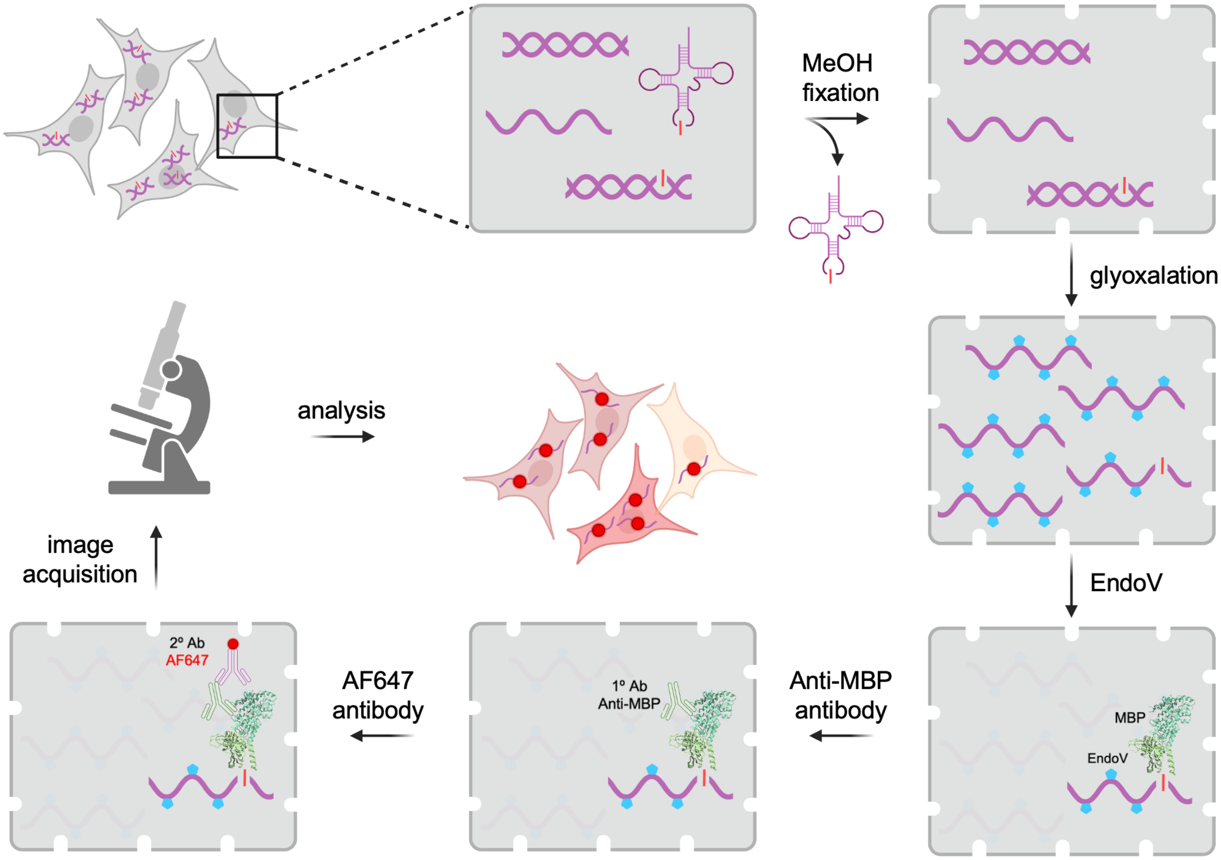

Figure S5. Summary of EndoVIA workflow. Cultured cells are fixed and permeabilized with 100% ice cold methanol followed by washing to remove tRNA. Fixed cells are then treated with a 12% glyoxal solution (blue hexagons) to denature RNA secondary structure. Following subsequent permeabilization and blocking steps, cells are incubated with a 1:50 EndoV calcium-containing solution to bind inosine-containing transcripts. Following EndoV treatment, cells are incubated with a 1:1000 anti-MBP antibody calcium-containing solution to bind the MBP tag fused to EndoV. Finally, cells are stained with a secondary antibody conjugated to Alexa Fluor 647 that is specific for the anti-MBP antibody. Cells are next imaged using fluorescent microscopy and analyzed.

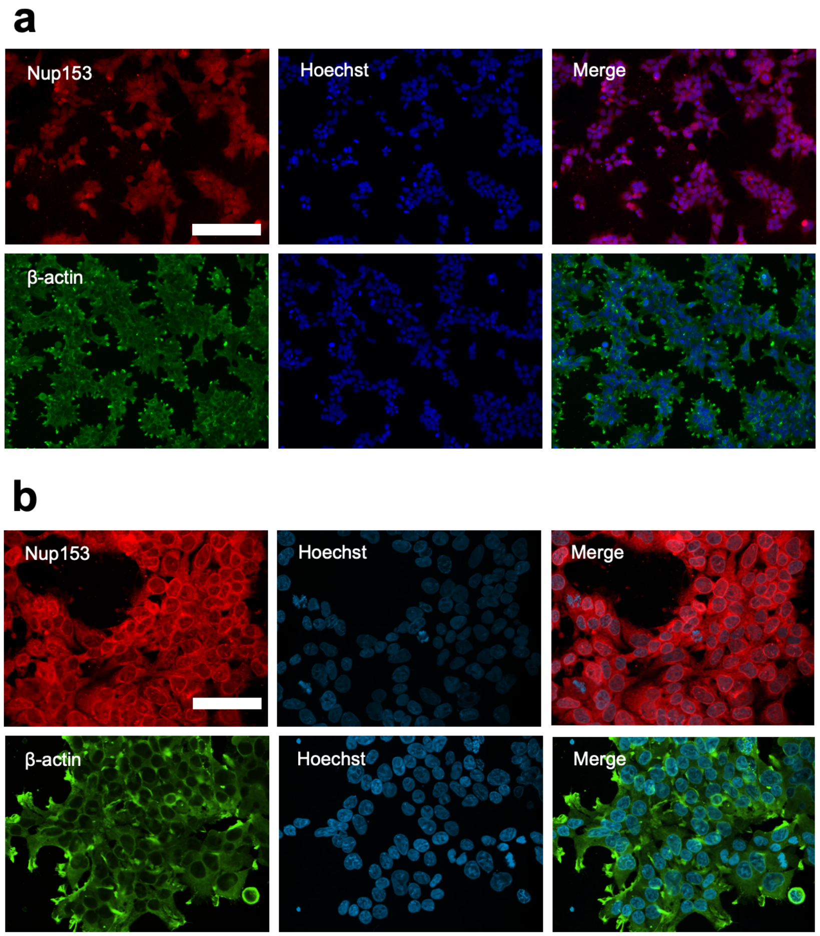

Figure S6. Detection of Nup153 and β-actin using modified immunofluorescence workflow. (a) HEK293T cells were fixed and stained for Nup153 (red) or β-actin (green) and cell nuclei (blue). Images were taken using widefield microscopy. (b) HEK293T cells were prepared as previously described in (b) and imaged using confocal microscopy. Data are representative of three independent experiments; *n=3* wells from a 96-well plate. Scale bar, (a) 200 µm and (b) 50 µm.

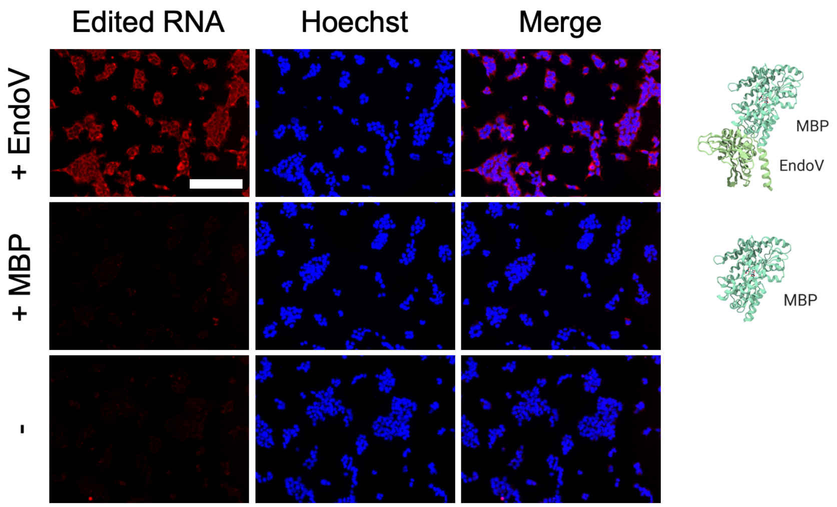

Figure S7. Non-specific binding of MBP of the EndoV-MBP fusion protein. HEK293T cells were fixed and stained for edited RNA (red) and cell nuclei (blue) using EndoV-MBP fusion protein (+ EndoV), MBP alone (+ MBP), or neither (-). Data are representative of three independent experiments; *n=3* wells from a 96-well plate. Scale bar, 200 μm.

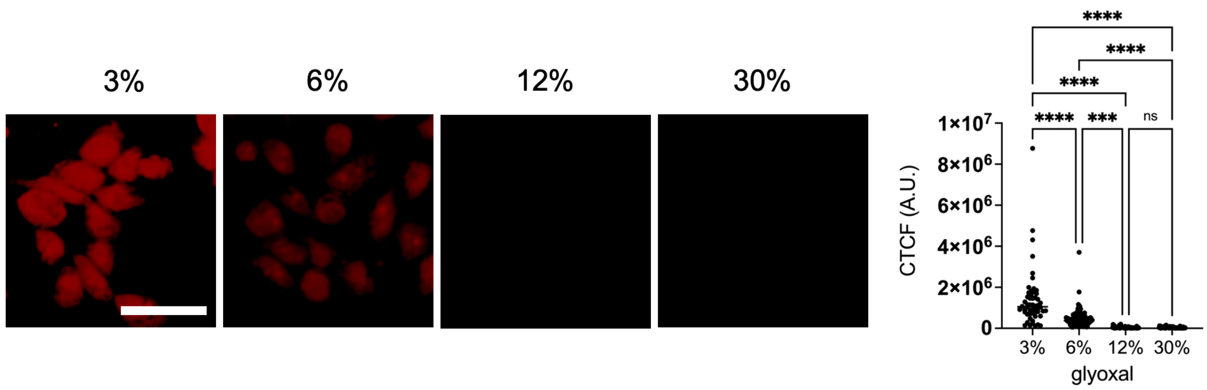

Figure S8. Optimizing glyoxal concentration using *GAPDH* fluorescence *in situ* hybridization (FISH). HEK293T cells were fixed and treated with increasing amounts of glyoxal (3%, 6%, 12%, 30%) and stained for *GAPDH* mRNA using FISH probes (red) and quantified for mean corrected total cellular fluorescence (CTCF). Data are representative of three independent experiments; *n=50* cells. Scale bar, 50 µm. Data are shown as CTCF per cell in arbitrary units (A.U.). Statistical significance was determined by one-way ANOVA; not significant (ns), ***$P < 0.001$, ****$P < 0.0001$.

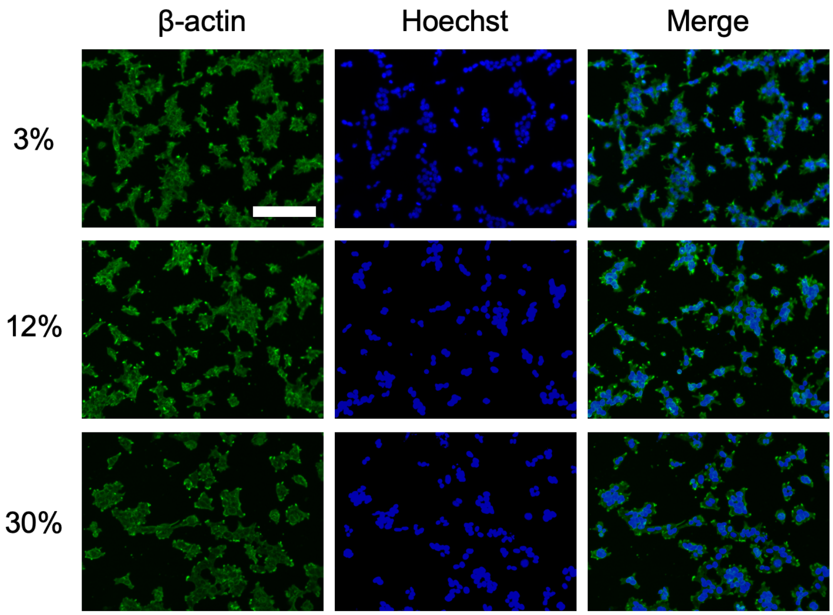

Figure S9. Cellular morphology of cells treated with varying glyoxal concentrations using β-actin. HEK293T cells were fixed and treated with increasing amounts of glyoxal (3%, 12%, 30%) and stained for β-actin (green) and cell nuclei (blue). Data are representative of three independent experiments. Scale bar, 200 μm.

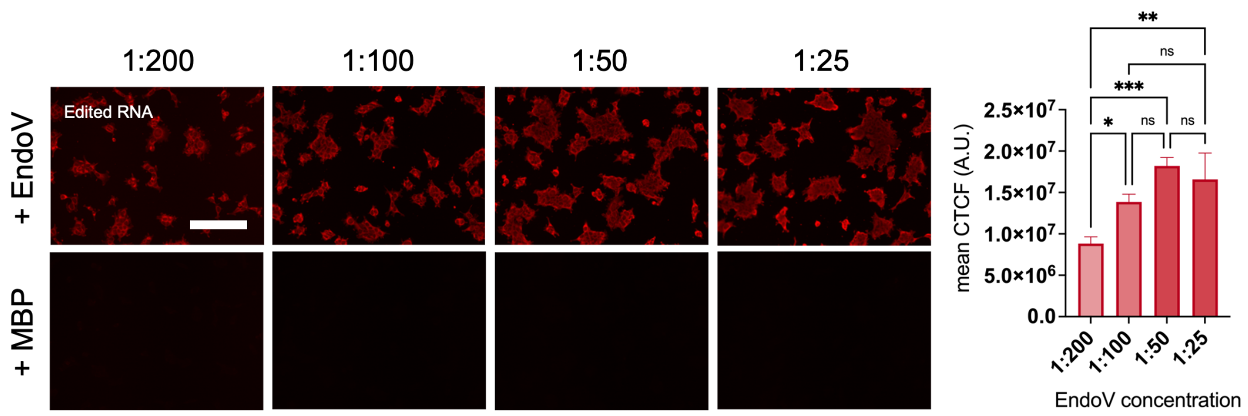

Figure S10. Optimizing EndoV concentration. HEK293T cells were fixed and stained for edited RNA (red) with increasing amounts of EndoV or MBP and quantified for mean corrected total cellular fluorescence (CTCF). Data are representative of three independent experiments; *n=3* wells from a 96-well plate. Scale bar, 200 µm. Data are shown as mean ± s.d. in arbitrary units (A.U.). Statistical significance was determined by one-way ANOVA; not significant (ns), **P* < 0.05, ***P* < 0.01, ****P* < 0.001.

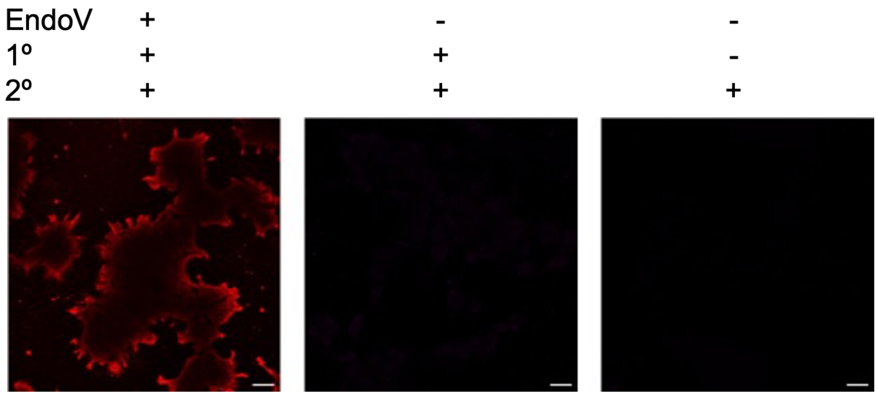

Figure S11. Antibody negative control for confocal imaging. HEK293T cells were fixed and stained using EndoVIA with (+) or without (-) EndoV, primary, and secondary antibody. Data are representative of three independent experiments; *n=3* coverslips. Scale bar, 10 µm.

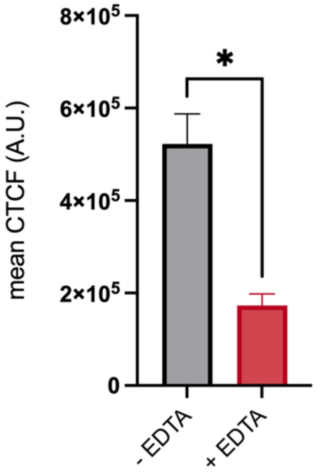

Figure S12. EndoV stained cells treated with EDTA. HEK293T cells were fixed and stained for edited RNA, treated with EDTA, and quantified for mean corrected total cellular fluorescence (CTCF). Data are representative of three independent experiments; *n=3* wells from a 96-well plate. Data are shown as mean ± s.d. in arbitrary units (A.U.). Statistical significance was determined by unpaired *t*-test; *$P < 0.05$.

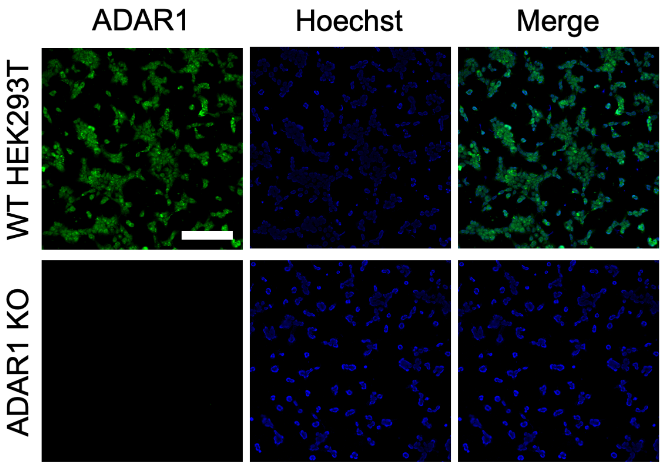

Figure S13. Immunostaining ADAR1. WT HEK293T cells and ADAR1 KO cells were fixed and stained for ADAR1. Data are representative of three independent experiments; *n=3* wells from a 96-well plate. Scale bar, 200 μm.

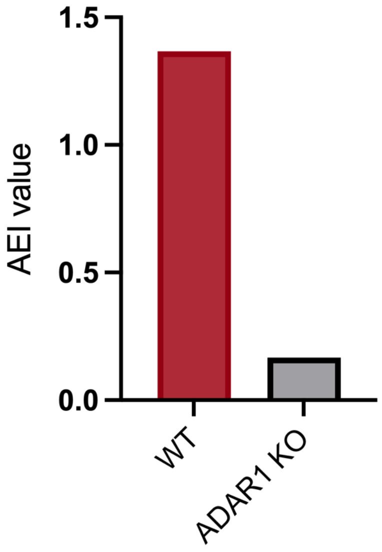

Figure S14. AEI Values of WT and ADAR1 KO HEK293T cells. Total RNA was isolated, purified, and sequenced. Resulting datasets were then trimmed, aligned, and sorted to calculate the AEI values.

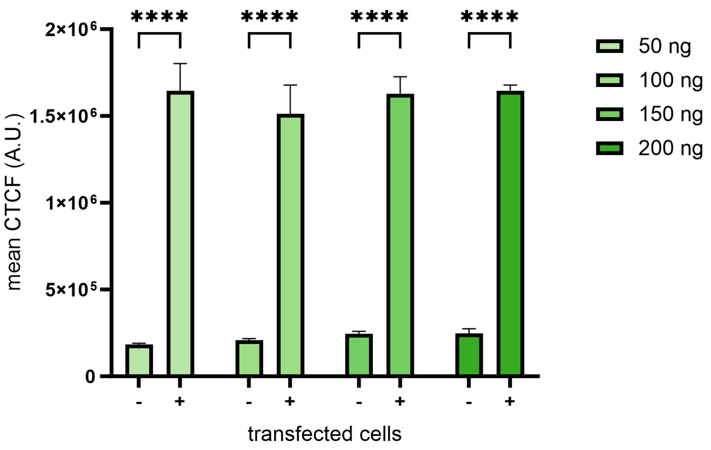

Figure S15. Detecting an increase in A-to-I editing in HEK293T cells. HEK293T cells were transfected with increasing amounts of GFP tagged ADAR1 p150 plasmid (0-200 ng), fixed, and stained for edited RNA (red) using EndoVIA. Quantification of mean corrected total cellular fluorescence (CTCF) of edited RNA was performed in ADAR-GFP positive (+) and negative (-) cells. Data are representative of three independent experiments; *n=3* wells from a 96-well plate. Data are shown as mean ± s.d. in arbitrary units (A.U.). Statistical significance was determined by unpaired *t*-test; ****$P < 0.0001$.

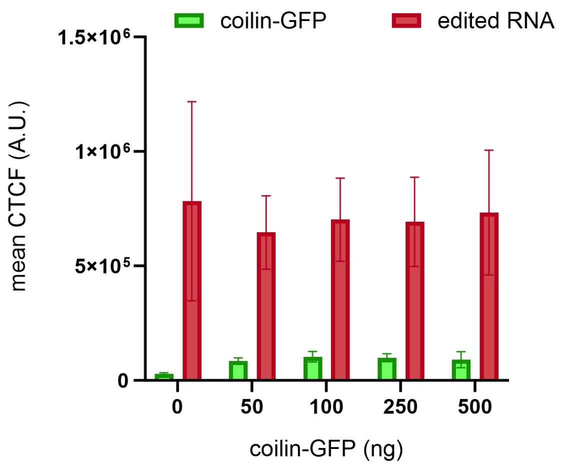

Figure S16. Quantifying A-to-I editing in HEK293T cells transfected with coilin-GFP. HEK293T cells were transfected with increasing amounts of coilin-GFP plasmid (0-500 ng), fixed, and stained for edited RNA (red) using EndoVIA. Coilin-GFP and edited RNA fluorescence were quantified for mean corrected total cellular fluorescence (CTCF). Data are representative of three independent experiments; *n=3* wells from a 96-well plate. Data are shown as mean ± s.d. in arbitrary units (A.U.).

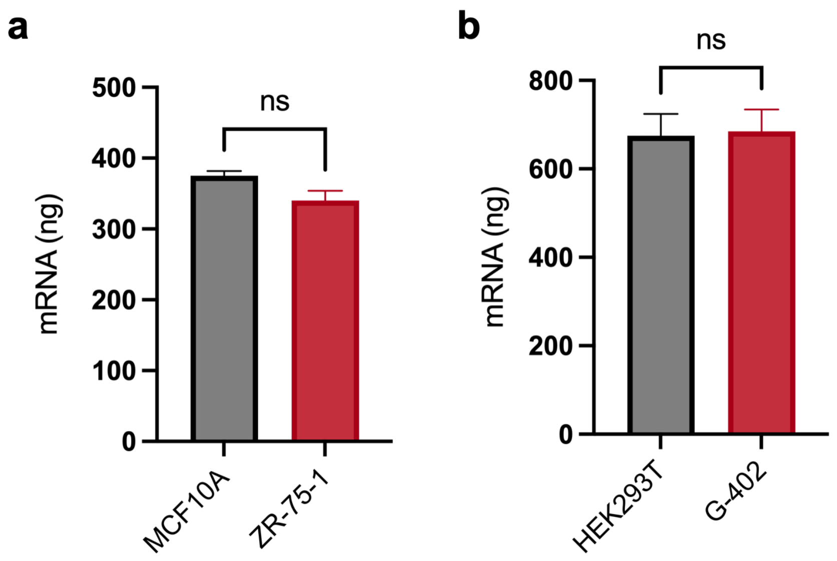

Figure S17. Quantifying mRNA in non-malignant and malignant cell lines. The mRNA from (a) breast cell lines MCF10A and ZR-75-1 and (b) kidney cell lines HEK293T and G-402 were isolated and quantified. Data are representative of three independent experiments; *n=3* wells from a 6-well plate. Data are shown as mean ± s.d. Statistical significance was determined by unpaired *t*-test; not significant (ns).

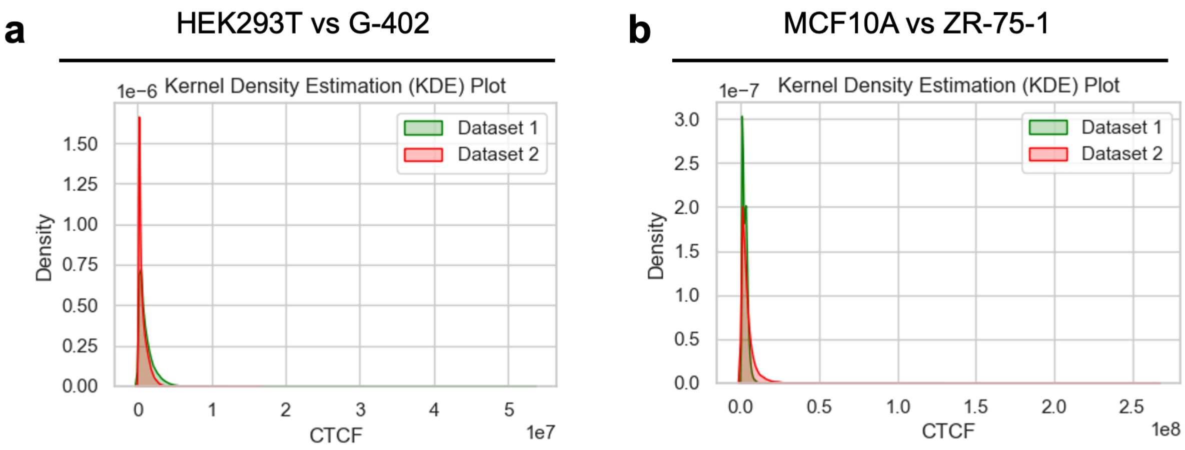

Figure S18. Cellular heterogeneity in non-malignant and malignant cell lines. The kernel density estimation was determined for (a) HEK293T (green) and G-402 (red) cells and (b) MCF10A (green) and ZR-75-1 (red) cells using the CTCF values of each individual cell. Data are representative of three independent experiments; *n=9* wells from a 96-well plate.

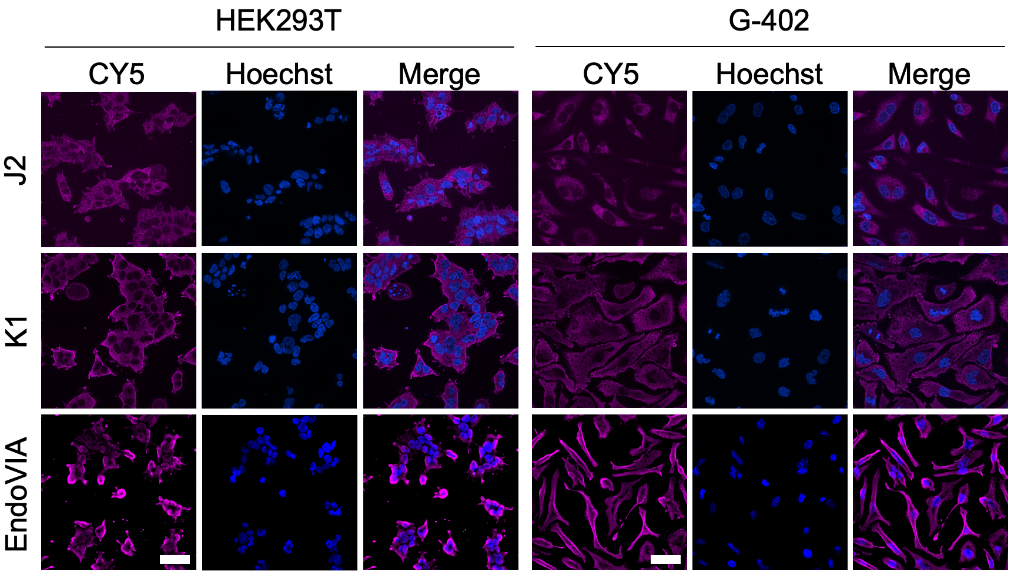

Figure S19. Immunostaining dsRNA and edited RNA in healthy and diseased cells. Fixed HEK293T and G-402 cells were immunostained for dsRNA (J2, K1) or edited RNA (EndoVIA). Data are representative of three independent experiments; *n=3* wells from a 96-well plate. Scale bar, 50 μm.

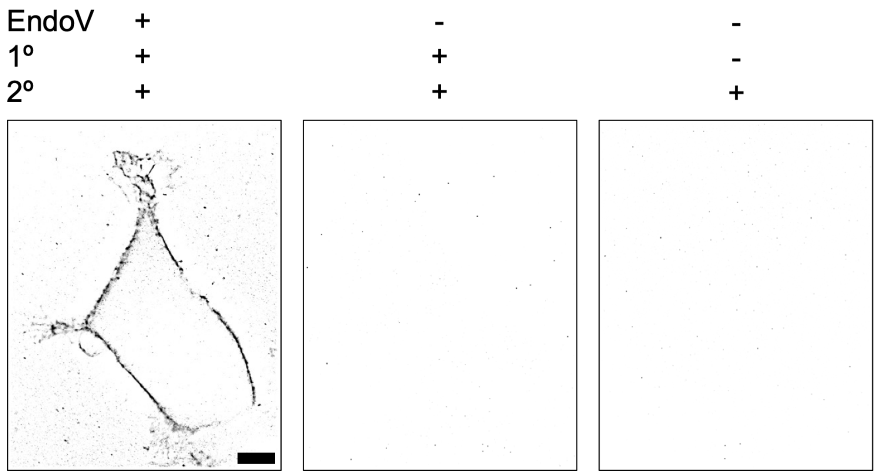

Figure S20. Antibody negative control for dSTORM in TIRF illumination imaging. HEK293T cells were fixed and stained using EndoVIA with (+) or without (-) EndoV, primary, and secondary antibody. Data are representative of three independent experiments; *n=3* coverslips. Scale bar, 10 µm.

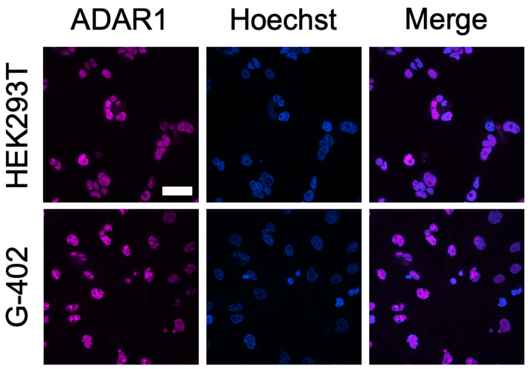

Figure S21. Immunostaining ADAR1 in HEK293T and G-402 cells. Fixed HEK293T and G-402 cells were immunostained for ADAR1. Data are representative of three independent experiments; *n=3* wells from a 96-well plate. Scale bar, 50 μm.
