# Supplementary material for: Spatial Visualization of A-to-I Editing in Cells Using Endonuclease V Immunostaining Assay (EndoVIA)
Source: ACS Cent Sci. 2024 Jul 8;10(7):1396–405. doi: 10.1021/acscentsci.4c00444 (PMC11273454; doi:10.1021/acscentsci.4c00444)
Supplement: Supplementary file 2 — oc4c00444_si_002.pdf [file oc4c00444_si_002.pdf]

oc-2024-00444u.R1

Name: Peer Review Information for "Spatial Visualization of A-to-I Editing in Cells using Endonuclease V Immunostaining Assay (EndoVIA)"

First Round of Reviewer Comments

Reviewer: 1

Comments to the Author

Quillin et. al. present EndoVIA, a method applying Endonuclease V Immunostaining to spatially quantify A-to-I editing through imaging, achieving higher resolution by employing super-resolution microscopy. The application of Endonuclease V for inosine recognition has been reported by the same group (Angew. Chem. 60, 17009–17017, 2021), with HRP-ligated secondary antibody and chemiluminescent detection, decreasing the novelty of EndoVIA. In addition, several key concerns also need to be addressed.

1. The authors describe the use of Endonuclease V for inosine imaging. Considering the availability of commercial inosine antibodies (MBL PM098 and Diagenode C1520025), which may also be used for inosine immunostaining. The authors should compare the specificity and efficiency between Endonuclease V and these antibodies for inosine imaging.

2. In Figure S1, the tRNA looks clearly cytoplasmic localized with formaldehyde fixation. Switching to methanol fixation, however, the tRNA signal exhibits as nuclear puncta, as well as nucleoplasm. What caused this shift towards nucleus under methanol fixation conditions? Given the much higher abundance of tRNA A-to-I editing compared to mRNA in mammals, it is crucial to assess impacts from tRNA on the accuracy of inosine detection.

3. Figure 3 indicates a ~50% reduction in inosine signal in ADAR1 KO cells versus WT in HEK293T, whereas RNA seq showed an ~85% decrease (AEI 1.4 to 1.2). The authors should address whether the 35% difference could be attributed to background signal or not. If yes, how would this influence the accuracy of imaging quantification?

4. The authors showed the inosine signal predominantly showed in cytoplasm near cell membrane. Can the authors verify this observation by an independent method without relying on Endonuclease V, such as cell fractionation followed by mass spectrometry or RNA seq?

5. In Figure 4, a substantial portion of cells failed to express ADAR1-GFP even at the highest amount of plasmid concentration. Are there differences in inosine signals between cells that express ADAR1-GFP and those do not within the same well?

6. In the upper panel of Figure 3a, the Hoechst staining differs from the merged image.

Reviewer: 2

Comments to the Author

Summary:

A-to-I editing is a ubiquitous RNA modification critical for development, immunity, and manipulated in disease. Modification in RNAs is performed in part by the ADAR family of enzymes. Much of the work regarding ADARs and biology has focused on genomics methods, mostly due to the lack of means to visualize modified RNAs in cells. Here, Quillin, et al. extend their work using an inosine binding enzyme as a molecular tool to visualize modified RNAs in cells. This manuscript shows how EndoVIA can label RNAs after chemical treatment. The manuscript reports a loss of EndoVIA signal in an ADAR1 KO cell line and EndoVIA images after plasmid expression of ADAR1. Prior work had identified cancer cell lines with hypo- and hyper- A-to-I editing. The manuscript confirms these results and shows that EndoVIA imaging levels correlate with the genomics. Super-resolution microscopy demonstrates the potential to use EndoVIA as a method to visualize modified RNA localization in cells.

The manuscript is well written and is a logical extension of the authors' previous work applying a cleaver chemical probe to study inosine RNA modifications. The general concern, however, is the modest differences in imaging values between the test and control samples. The body of work implies that EndoVIA consistently can measure inosine signal over noise, but the quantified values suggest that this method is a half-step away from its limits of detection. The contribution of tRNA signal remains a concern when trying to decipher transcript signal. Regardless, there are very few methods available to visualize RNA modifications in cells and in vivo. As such, the work will be of general interest to RNA biologists, cell biologists, and those studying health and disease processes that involve ADAR and A-to-I editing.

MAJOR

1. A workflow diagram outlining EndoVIA cell imaging, from start to finish that includes key chemical details (e.g. glyoxal treatment), will be helpful to summarize the method.

2. “We also observed a direct relationship between the fluorescence of ADAR-GFP expression and edited RNA (Figure 4b).” Without statistical comparisons between the data, it is not clear how this conclusion was reached. By eye, the EndoVIA fluorescent levels look about the same. ADAR fluorescent levels also did not correlate with plasmid transfected. Was there an attempt to quantify EndoVIA signal in just GFP fluorescent cells? This analysis may be more accurate in measuring the success of the system and this particular experiment.

3. The super resolution images presented in Fig 6 are striking but have caveats. There are no negative controls presented (EndoVIA minus with just secondary Ab) for these two imaging modalities. Additionally, the manuscript implies that we are observing subcellular localization of modified transcripts. The image presented in Fig S1 suggests that methanol decreases but does not entirely remove the presence of tRNAs from cells. Thus, there is a high likelihood that at least some of this signal is due to tRNAs. Note that this second point is true for all of the images shown. The manuscript text should clarify what we may be looking at or present additional controls accounting for the tRNA “background.”

MINOR:

1. While very well written and informative, the introduction and first 2 figures currently read more as a comprehensive ADAR review rather than background for the manuscript presented. Some of the introduction could have been summarized (e.g. 3’UTR examples on page 3). This reader could have also used a (brief) summary on general RNA imaging strategies and how these strategies are generally not equipped to detect RNA modifications.

2. Much of the development and optimization sections were also well written but incredibly detailed. Moving much of this detail into the methods section would help streamline the manuscript.

3. Summarizing work imaging RNA modifications and their discoveries may be helpful to justify the uses of EndoVIA that the manuscript proposes in the discussion.

Author's Response to Peer Review Comments:

Jennifer M. Heemstra, PhD
Charles Allen Thomas Professor
Chair, Department of Chemistry
heemstra@wustl.edu
314-935-4269

May 9, 2024

Deputy Editor
ACS Central Science

Dear Editor,

Thank you for considering a revised version of the manuscript oc-2024-00444u entitled "Spatial Visualization of A-to-I Editing in Cells using Endonuclease V Immunostaining Assay (EndoVIA)" for publication as an article in *ACS Central Science*.

We have addressed the comments and concerns of the reviewers by performing additional experiments and analyses to strengthen the arguments presented in our manuscript. We have also modified the manuscript to reflect these new experiments and to improve clarity and conciseness. Included with this cover letter is a list that details our responses and changes to the manuscript. We feel that these changes significantly improve the manuscript, and we thank the reviewers for their comments.

We hope that we have addressed all of the concerns with this manuscript, and we thank you for your time. Feel free to contact us if you have any questions or require further information.

Sincerely,

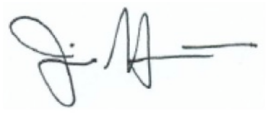

Jennifer M. Heemstra, Ph.D.
Charles Allen Thomas Professor
Chair, Department of Chemistry

Washington University in St. Louis, MSC 1134-196-525, One Brookings Drive, St. Louis, Missouri 63130-4899
Department Phone: (314) 935-6530 http://www.chemistry.wustl.edu

RESPONSE TO REVIEWER'S COMMENTS

REVIEWER 1

Reviewer Comment: The application of Endonuclease V for inosine recognition has been reported by the same group (Angew. Chem. 60, 17009–17017, 2021), with HRP-ligated secondary antibody and chemiluminescent detection, decreasing the novelty of EndoVIA.

Response: We thank the reviewer for this comment. While our previous methods have utility for studying pooled RNA extracted from cells, they are not able to image and quantify editing *in situ*. While beyond the scope of this initial report, such capability will enable experiments such as high-throughput phenotypic drug screening. We have included a short description in the Introduction that highlights these key points and justifies the novelty of the current work:

While our group's previous efforts have leveraged EndoV to accurately quantify A-to-I editing, these methods still require RNA extraction and pooling of RNA from a population of cells. These steps effectively erase information regarding the spatial distribution of edited RNA *in situ* as well as variation in editing signatures between cells. Seeking to overcome the limitations of these *in vitro* approaches, we recognized that the inosine binding capabilities of EndoV could be harnessed for detecting A-to-I edited transcripts *in situ* to retain RNA localization and cellular heterogeneity by recapitulating the principles of immunofluorescence (Figure 2c).

Reviewer Comment: The authors describe the use of Endonuclease V for inosine imaging. Considering the availability of commercial inosine antibodies (MBL PM098 and Diagenode C1520025), which may also be used for inosine immunostaining. The authors should compare the specificity and efficiency between Endonuclease V and these antibodies for inosine imaging.

Response: We agree that this is an important comparison. While the general knowledge in the field is that these antibodies do not have the affinity needed for sensitive detection of inosine, it is important to demonstrate this in our manuscript. We first performed immunostaining with the MBL inosine antibody (PM098) and Diagenode inosine antibody (C1520025) and compared these results with EndoV. Imaging revealed that the MBL antibody displayed high levels of nuclear localization and the Diagenode antibody showed relatively low overall fluorescence in comparison to EndoV immunostaining. However, further optimization of antibody concentration and characterization would be required to draw definitive conclusions on the performance of these commercially available antibodies *in situ*.

In parallel, we also performed microscale thermophoresis (MST) to determine the specificity and affinity of these anti-inosine antibodies for edited (RNA I) and unedited (RNA A) transcripts. We determined that while the specificity of these antibodies for RNA I over RNA A are comparable to that of EndoV, the K_d values for the Diagnode and MBL antibodies are 87.4 nM and 602 nM, respectively, which reflects orders of magnitude weaker binding than EndoV (K_d = 3.4 nM). Thus, in our hands, these antibodies do not perform at a level that would be needed for our immunostaining application.

The MST data have been added to the Supplemental Information (Figure S1). While the immunostaining data strongly suggest that the antibodies would not be effective for this application, we are concerned about reporting these images without extensive optimization experiments. At the same time, the incremental improvement that would likely be realized from such optimizations does not seem worthwhile given what we observe via MST. Moreover, only one of these antibodies has been classified as being appropriate for immunostaining. Thus, while these images clearly demonstrate the need for EndoV as an improved affinity reagent for inosine-containing RNAs, we don't feel that it would reflect rigorous scholarship to include the images from the antibody comparisons in our manuscript. Thus, these images are only being included below for review purposes.

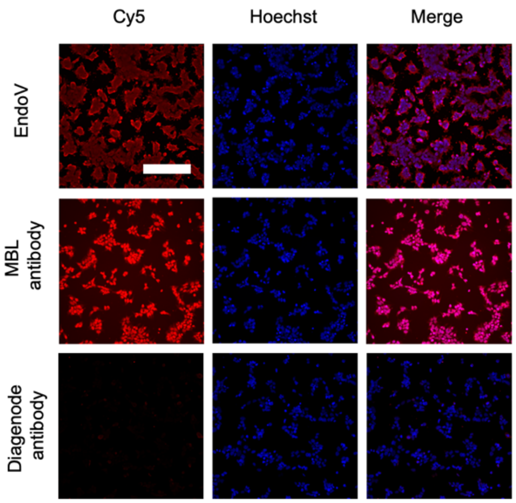

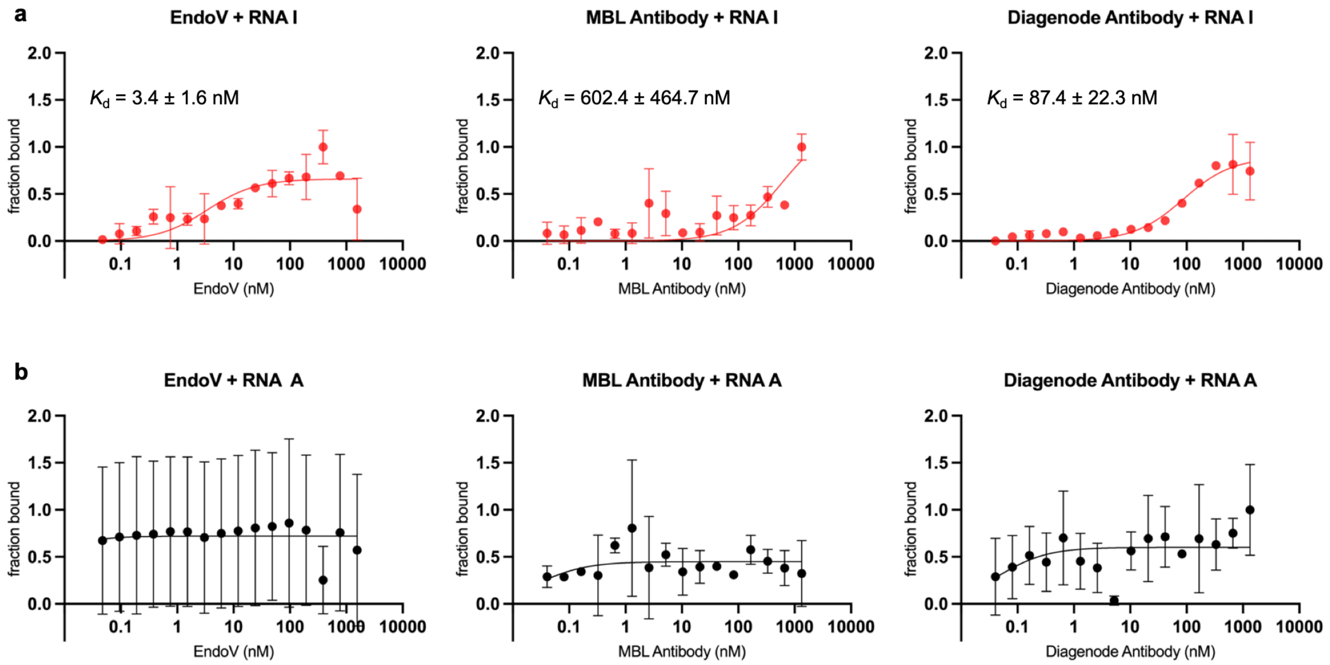

Reviewer Comment: In Figure S1, the tRNA looks clearly cytoplasmic localized with formaldehyde fixation. Switching to methanol fixation, however, the tRNA signal exhibits as nuclear puncta, as well as nucleoplasm. What caused this shift towards nucleus under methanol fixation conditions? Given the much higher abundance of tRNA A-to-I editing compared to mRNA in mammals, it is crucial to assess impacts from tRNA on the accuracy of inosine detection.

Response: We agree with the reviewer that assessing the impact of residual tRNA is important. We immunostained tRNA in both formaldehyde-fixed and methanol-fixed cells and determined that ~72% of tRNA is removed using methanol fixation and that the residual tRNA does appear to localize to nuclear puncta. These data have been included in Figure S3 and Figure S4. As noted in the comment below, the residual tRNA only seems to interfere with quantification in cell lines having very low editing, and we have added a note to this effect in the manuscript.

Reviewer Comment: Figure 3 indicates a ~50% reduction in inosine signal in ADAR1 KO cells versus WT in HEK293T, whereas RNA seq showed an ~85% decrease (AEI 1.4 to 1.2). The authors should address whether the 35% difference could be attributed to background signal or not. If yes, how would this influence the accuracy of imaging quantification?

Response: Based on the quantified tRNA remaining in cells after methanol fixation in the new Figure S4, we attribute the difference in WT and ADAR1 KO cells to tRNA background. The remaining ~28% of tRNA post methanol fixation can cause error in quantitation of relative ADAR editing levels for cells that have very low editing such as HEK293T. However, we note that this residual tRNA does not contribute significantly to error in more moderate editing cells such as the healthy and cancerous breast cell lines, as the quantified fluorescence via immunostaining is consistent with the ratio of AEI values determined by sequencing. These data lead us to believe that the overall background from tRNA is small. However, future work in our group will focus on developing affinity reagents that can bind inosine in dsRNA as this would further alleviate challenges from tRNA background, but those results are beyond the scope of the current manuscript.

Reviewer Comment: The authors showed the inosine signal predominantly showed in cytoplasm near cell membrane. Can the authors verify this observation by an independent method without relying on Endonuclease V, such as cell fractionation followed by mass spectrometry or RNA seq?

Response: We appreciate this comment but note that such fractionation would be extremely complicated, as methods do not exist for isolating molecules that are near to (but not embedded in) the membrane. We note that for other peer-reviewed publications reporting sub-cellular localization of biomolecules, the imaging experiments (with appropriate controls) generally suffice.

Reviewer Comment: In Figure 4, a substantial portion of cells failed to express ADAR1-GFP even at the highest amount of plasmid concentration. Are there differences in inosine signals between cells that express ADAR1-GFP and those do not within the same well?

Response: We are very grateful to the reviewer for this suggestion, as it dramatically improved our analysis of these data! We performed analysis as suggested and found that inosine fluorescence in ADAR1-GFP positive cells is significantly higher than ADAR1-GFP negative cells. We also purified new ADAR1-p110-GFP and ADAR1-p150-GFP plasmid to reattempt the transfection with a more suitable range of plasmid concentrations for a 96-well plate and performed the suggested analysis. We were encouraged to find that transfection yields improved and ADAR1-GFP positive cells showed significantly increased inosine fluorescence in comparison to ADAR1-GFP negative cells, consistent with the previously collected data and the reviewer's suggestion. These new data have been incorporated into the manuscript in Figure 4 and Figure S15 and the text has been updated.

Reviewer Comment: In the upper panel of Figure 3a, the Hoechst staining differs from the merged image.

Response: We thank the reviewer for noticing this oversight and have replaced the Hoechst image accordingly in Figure 3a.

REVIEWER 2

Reviewer Comment: A workflow diagram outlining EndoVIA cell imaging, from start to finish that includes key chemical details (e.g. glyoxal treatment), will be helpful to summarize the method.

Response: We agree that this would be helpful for researchers who wish to utilize our method. Thus, to address this feedback, we have included a new figure summarizing the EndoVIA workflow in the Supplemental Information (Figure S5).

Reviewer Comment: "We also observed a direct relationship between the fluorescence of ADAR-GFP expression and edited RNA (Figure 4b)." Without statistical comparisons between the data, it is not clear

how this conclusion was reached. By eye, the EndoVIA fluorescent levels look about the same. ADAR fluorescent levels also did not correlate with plasmid transfected. Was there an attempt to quantify EndoVIA signal in just GFP fluorescent cells? This analysis may be more accurate in measuring the success of the system and this particular experiment.

Response: We thank the reviewer for this feedback. In response to a similar comment and suggestion from Reviewer 1, we reanalyzed our data to specifically calculate the fluorescence of ADAR1-GFP positive vs negative cells and found a significant difference in EndoV immunostaining signal between these two populations. We also repeated the this experiment with newly purified ADAR1-p110-GFP and ADAR1-p150-GFP plasmid and more suitable concentrations for a 96-well plate and were encouraged to observe improved transfection and significant increase in inosine signal in ADAR1-GFP positive cells, consistent with the previous data. These new data have been incorporated into the manuscript in Figure 4 and Figure S15 and the text has been updated accordingly.

Reviewer Comment: The super resolution images presented in Fig 6 are striking but have caveats. There are no negative controls presented (EndoVIA minus with just secondary Ab) for these two imaging modalities.

Response: We agree and have completed two additional negative controls by performing EndoVIA omitting EndoV, as well as omitting EndoV and the primary antibody. We imaged using both confocal and dSTORM in TIRF illumination and observed no detectable fluorescence in the negative controls. We have included these data in the Supplementary Information in Figure S11 and Figure S20.

Reviewer Comment: Additionally, the manuscript implies that we are observing subcellular localization of modified transcripts. The image presented in Fig S1 suggests that methanol decreases but does not entirely remove the presence of tRNAs from cells. Thus, there is a high likelihood that at least some of this signal is due to tRNAs. Note that this second point is true for all of the images shown. The manuscript text should clarify what we may be looking at or present additional controls accounting for the tRNA “background.”

Response: We agree with the reviewer that some of the fluorescence comes from residual tRNA and that this needs to be accounted for. We determined that methanol fixation removes ~72% of tRNA and have included these data in Figure S4. While the remaining tRNA can cause error in quantitation of relative ADAR editing levels for cells that have very low editing such as HEK293T, we note that this residual tRNA it does not contribute significantly to error in more moderate editing cells such as the healthy and cancerous breast cell lines, as the quantified fluorescence via immunostaining is consistent with the ratio of AEI values determined by sequencing. These data lead us to believe that the overall background signal from tRNA is small.

Encouragingly, while tRNA FISH reveals that the residual tRNA is primarily located in nuclear foci, we do not observe significant signal with this localization pattern in our single-molecule imaging experiments. This suggests that the relative contribution of this tRNA is indeed very minor. We have added a note to the manuscript outlining these caveats and suggesting that tRNA FISH be performed as a control in order to identify any background signal arising from the inherently incomplete removal of tRNA:

We do note that some residual tRNA can be present after the fixation steps and may lead to background signal. However, tRNA FISH in the HEK293T cells reveals that that this remaining tRNA is primarily sequestered to nuclear foci (Figure S3), and we do not observe a strong corresponding EndoV signal in our single-molecule EndoVIA experiments. This suggests that the interference of residual tRNA is minimal, though out of an abundance of caution we do recommend that tRNA FISH be included as a control experiment when using EndoVIA to map subcellular localization of edited RNAs.

Reviewer Comment: While very well written and informative, the introduction and first 2 figures currently read more as a comprehensive ADAR review rather than background for the manuscript presented. Some of the introduction could have been summarized (e.g. 3’UTR examples on page 3). This reader could have also used a (brief) summary on general RNA imaging strategies and how these strategies are generally not equipped to detect RNA modifications.

Response: We thank the reviewer for this feedback and have revised the introduction accordingly by removing redundant information in the paragraph regarding ADAR and innate cellular immunity, as well as summarizing the paragraph describing editing within 3' UTRs. A paragraph was also included that summarizes traditional RNA imaging techniques and specialized methods for imaging RNA modifications.

Reviewer Comment: Much of the development and optimization sections were also well written but incredibly detailed. Moving much of this detail into the methods section would help streamline the manuscript.

Response: To address this feedback we have moved multiple portions from the sections "Development of EndoVIA for *in situ* Imaging of A-to-I Edited Transcripts" and "Optimization of EndoVIA" into the Methods section located in the Supplementary Information.

Reviewer Comment: Summarizing work imaging RNA modifications and their discoveries may be helpful to justify the uses of EndoVIA that the manuscript proposes in the discussion.

Response: We thank the reviewer for this suggestion and have incorporated a paragraph in the Introduction that summarizes current approaches for imaging RNA modifications and their contributions to the field:

Traditional cellular RNA imaging techniques, such as fluorescence *in situ* hybridization (FISH) and genetically encodable tags that bind fluorescent proteins or dyes, have revolutionized the way RNA is visualized in cells.[29–31] However, these approaches alone cannot detect editing status and lack the sensitivity required to discern single nucleotide modifications. As a result, specialized methods have been developed to image the localization of RNA modifications. Some of the first examples are FISH-inspired methods that leverage toehold probes to detect single nucleotide variants (SNVs) in RNA, including the first demonstration of visualizing A-to-I editing status of specific transcripts using inosine fluorescence *in situ* hybridization (inoFISH) thus providing insights into the link between editing patterns and localization.[32,33] Approaches have also been designed to detect cytidine-to-uridine (C-to-U) editing of RNAs of interest in cells using forced intercalation probes (FIT) probes.[34,35] A generalizable method for targeting polyadenylated RNAs coined click-encoded rolling FISH (ClickerFISH) was also developed and allowed the visualization of higher-order structures at the subcellular level with cell-to-cell.[36] Most recently, deamination adjacent to RNA modification targets FISH (DART-FISH) was developed to enable *in situ* detection of individual m^6A-modified and unmodified transcripts and determined that m^6A alone is not sufficient to localize mRNA to stress granules.[37] Together, these select examples highlight the importance of novel techniques to reveal the impact RNA modifications play intracellularly. Despite these advances that target specific transcripts, the need remains for a generalizable approach for quantifying and mapping the localization of inosine-containing RNAs across the transcriptome.[33] Antibodies could prove advantageous towards this goal, yet efforts to generate an anti-inosine antibody have fallen short, with the resulting affinity reagents lacking the affinity and reproducibility needed for rigorous analytical methods (Figure S1).[38] Taken together, a method for detecting and quantifying A-to-I edited RNA *in situ* remains an unmet challenge.

RESPONSE TO EDITOR'S COMMENTS

Comment: MS File: References truncated using "et al." References do not have more than one author name included in the standard format.

Response: We have updated the references to reflect this formatting requirement.

Comment: MS File: Citations Not Numbered

Response: We have numbered the citations accordingly.

oc-2024-00444u.R2

Name: Peer Review Information for "Spatial Visualization of A-to-I Editing in Cells using Endonuclease V Immunostaining Assay (EndoVIA)"

First/Second/Third Round of Reviewer Comments

Reviewer: 1

Comments to the Author

The authors have added more experiments and addressed my concerns.

Reviewer: 2

Comments to the Author

I thank the authors for their efforts in the revisions. My previous comments have been addressed. A minor comment is that some of the figure text is very small. For example, the label text in Fig S5 is probably close to 2 pt font and will be challenging to read if printed.

Author's Response to Peer Review Comments:

Jennifer M. Heemstra, PhD
Charles Allen Thomas Professor
Chair, Department of Chemistry
heemstra@wustl.edu
314-935-4269

June 19, 2024

Deputy Editor
ACS Central Science

Dear Editor

We are excited to hear that our manuscript oc-2024-00444u entitled “Spatial Visualization of A-to-I Editing in Cells using Endonuclease V Immunostaining Assay (EndoVIA)” may be suitable for publication in *ACS Central Science* pending the requested revisions.

We have updated the figures in the manuscript and supplemental information with increased font size and addressed the formatting requirements. Included with this cover letter is a list that details these changes to the manuscript.

We hope that we have addressed all of the concerns with this manuscript, and we thank you for your time. Feel free to contact us if you have any questions or require further information.

Sincerely,

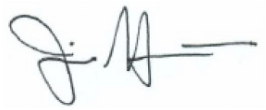

Jennifer M. Heemstra, Ph.D.
Charles Allen Thomas Professor
Chair, Department of Chemistry

Washington University in St. Louis, MSC 1134-196-525, One Brookings Drive, St. Louis, Missouri 63130-4899
Department Phone: (314) 935-6530 http://www.chemistry.wustl.edu

RESPONSE TO REVIEWER'S COMMENTS

REVIEWER 2

Reviewer Comment: I thank the authors for their efforts in the revisions. My previous comments have been addressed. A minor comment is that some of the figure text is very small. For example, the label text in Fig S5 is probably close to 2 pt font and will be challenging to read if printed.

Response: We agree with the reviewer that many of our font sizes were too small to be easily read, and have increased the font size of the text and figure labels in the majority of figures within the main text and supplementary information.

RESPONSE TO EDITOR'S COMMENTS

Comment: SI PARAGRAPH: If the manuscript is accompanied by any supporting information for publication, a brief description of the supplementary material is required in the manuscript. The appropriate format is: Supporting Information. Brief statement in non-sentence format listing the contents of the material supplied as Supporting Information.

Response: We have updated the Supporting Information section accordingly:

Experimental methods, sources of materials, and nucleic acid sequences, as well as Figures S1-S21 (PDF)
